# Sex-specific genetic risks for adverse outcomes after coronary revascularization procedures

**DOI:** 10.1093/icvts/ivae006

**Published:** 2024-01-12

**Authors:** Jouko Marko Nurkkala, Jenni Aittokallio, FinnGen, Aarno Palotie, Mark Daly, Bridget Riley-Gills, Howard Jacob, Dirk Paul, Athena Matakidou, Adam Platt, Heiko Runz, Sally John, George Okafo, Nathan Lawless, Heli Salminen-Mankonen, Robert Plenge, Joseph Maranville, Mark McCarthy, Julie Hunkapiller, Margaret G Ehm, Kirsi Auro, Simonne Longerich, Caroline Fox, Anders Mälarstig, Katherine Klinger, Deepak Raipal, Eric Green, Robert Graham, Robert Yang, Chris ÓDonnell, Tomi P Mäkelä, Jaakko Kaprio, Petri Virolainen, Antti Hakanen, Terhi Kilpi, Markus Perola, Jukka Partanen, Anne Pitkäranta, Taneli Raivio, Raisa Serpi, Tarja Laitinen, Veli-Matti Kosma, Jari Laukkanen, Marco Hautalahti, Outi Tuovila, Raimo Pakkanen, Jeffrey Waring, Bridget Riley-Gillis, Fedik Rahimov, Ioanna Tachmazidou, Chia-Yen Chen, Heiko Runz, Zhihao Ding, Marc Jung, Shameek Biswas, Rion Pendergrass, Julie Hunkapiller, Margaret G Ehm, David Pulford, Neha Raghavan, Adriana Huertas-Vazquez, Jae-Hoon Sul, Anders Mälarstig, Xinli Hu, Katherine Klinger, Robert Graham, Eric Green, Sahar Mozaffari, Dawn Waterworth, Nicole Renaud, Máen Obeidat, Samuli Ripatti, Johanna Schleutker, Markus Perola, Mikko Arvas, Olli Carpén, Reetta Hinttala, Johannes Kettunen, Arto Mannermaa, Katriina Aalto-Setälä, Mika Kähönen, Jari Laukkanen, Johanna Mäkelä, Reetta Kälviäinen, Valtteri Julkunen, Hilkka Soininen, Anne Remes, Mikko Hiltunen, Jukka Peltola, Minna Raivio, Pentti Tienari, Juha Rinne, Roosa Kallionpää, Juulia Partanen, Ali Abbasi, Adam Ziemann, Nizar Smaoui, Anne Lehtonen, Susan Eaton, Heiko Runz, Sanni Lahdenperä, Shameek Biswas, Julie Hunkapiller, Natalie Bowers, Edmond Teng, Rion Pendergrass, Fanli Xu, David Pulford, Kirsi Auro, Laura Addis, John Eicher, Qingqin S Li, Karen He, Ekaterina Khramtsova, Neha Raghavan, Martti Färkkilä, Jukka Koskela, Sampsa Pikkarainen, Airi Jussila, Katri Kaukinen, Timo Blomster, Mikko Kiviniemi, Markku Voutilainen, Mark Daly, Ali Abbasi, Jeffrey Waring, Nizar Smaoui, Fedik Rahimov, Anne Lehtonen, Tim Lu, Natalie Bowers, Rion Pendergrass, Linda McCarthy, Amy Hart, Meijian Guan, Jason Miller, Kirsi Kalpala, Melissa Miller, Xinli Hu, Kari Eklund, Antti Palomäki, Pia Isomäki, Laura Pirilä, Oili Kaipiainen-Seppänen, Johanna Huhtakangas, Nina Mars, Ali Abbasi, Jeffrey Waring, Fedik Rahimov, Apinya Lertratanakul, Nizar Smaoui, Anne Lehtonen, Marla Hochfeld, Natalie Bowers, Rion Pendergrass, Jorge Esparza Gordillo, Kirsi Auro, Dawn Waterworth, Fabiana Farias, Kirsi Kalpala, Nan Bing, Xinli Hu, Tarja Laitinen, Margit Pelkonen, Paula Kauppi, Hannu Kankaanranta, Terttu Harju, Riitta Lahesmaa, Nizar Smaoui, Glenda Lassi, Susan Eaton, Hubert Chen, Rion Pendergrass, Natalie Bowers, Joanna Betts, Kirsi Auro, Rajashree Mishra, Majd Mouded, Debby Ngo, Teemu Niiranen, Felix Vaura, Veikko Salomaa, Kaj Metsärinne, Jenni Aittokallio, Mika Kähönen, Jussi Hernesniemi, Daniel Gordin, Juha Sinisalo, Marja-Riitta Taskinen, Tiinamaija Tuomi, Timo Hiltunen, Jari Laukkanen, Amanda Elliott, Mary Pat Reeve, Sanni Ruotsalainen, Benjamin Challis, Dirk Paul, Julie Hunkapiller, Natalie Bowers, Rion Pendergrass, Audrey Chu, Kirsi Auro, Dermot Reilly, Mike Mendelson, Jaakko Parkkinen, Melissa Miller, Tuomo Meretoja, Heikki Joensuu, Olli Carpén, Johanna Mattson, Eveliina Salminen, Annika Auranen, Peeter Karihtala, Päivi Auvinen, Klaus Elenius, Johanna Schleutker, Esa Pitkänen, Nina Mars, Mark Daly, Relja Popovic, Jeffrey Waring, Bridget Riley-Gillis, Anne Lehtonen, Jennifer Schutzman, Julie Hunkapiller, Natalie Bowers, Rion Pendergrass, Diptee Kulkarni, Kirsi Auro, Alessandro Porello, Andrey Loboda, Heli Lehtonen, Stefan McDonough, Sauli Vuoti, Kai Kaarniranta, Joni A Turunen, Terhi Ollila, Hannu Uusitalo, Juha Karjalainen, Esa Pitkänen, Mengzhen Liu, Heiko Runz, Stephanie Loomis, Erich Strauss, Natalie Bowers, Hao Chen, Rion Pendergrass, Kaisa Tasanen, Laura Huilaja, Katariina Hannula-Jouppi, Teea Salmi, Sirkku Peltonen, Leena Koulu, Nizar Smaoui, Fedik Rahimov, Anne Lehtonen, David Choy, Rion Pendergrass, Dawn Waterworth, Kirsi Kalpala, Ying Wu, Pirkko Pussinen, Aino Salminen, Tuula Salo, David Rice, Pekka Nieminen, Ulla Palotie, Maria Siponen, Liisa Suominen, Päivi Mäntylä, Ulvi Gursoy, Vuokko Anttonen, Kirsi Sipilä, Rion Pendergrass, Hannele Laivuori, Venla Kurra, Laura Kotaniemi-Talonen, Oskari Heikinheimo, Ilkka Kalliala, Lauri Aaltonen, Varpu Jokimaa, Johannes Kettunen, Marja Vääräsmäki, Outi Uimari, Laure Morin-Papunen, Maarit Niinimäki, Terhi Piltonen, Katja Kivinen, Elisabeth Widen, Taru Tukiainen, Mary Pat Reeve, Mark Daly, Niko Välimäki, Eija Laakkonen, Jaakko Tyrmi, Heidi Silven, Eeva Sliz, Riikka Arffman, Susanna Savukoski, Triin Laisk, Natalia Pujol, Mengzhen Liu, Bridget Riley-Gillis, Rion Pendergrass, Janet Kumar, Kirsi Auro, Iiris Hovatta, Chia-Yen Chen, Erkki Isometsä, Hanna Ollila, Jaana Suvisaari, Thomas Damm Als, Antti Mäkitie, Argyro Bizaki-Vallaskangas, Sanna Toppila-Salmi, Tytti Willberg, Elmo Saarentaus, Antti Aarnisalo, Eveliina Salminen, Elisa Rahikkala, Johannes Kettunen, Kristiina Aittomäki, Fredrik Åberg, Mitja Kurki, Samuli Ripatti, Mark Daly, Juha Karjalainen, Aki Havulinna, Juha Mehtonen, Priit Palta, Shabbeer Hassan, Pietro Della Briotta Parolo, Wei Zhou, Mutaamba Maasha, Shabbeer Hassan, Susanna Lemmelä, Manuel Rivas, Mari E Niemi, Aarno Palotie, Aoxing Liu, Arto Lehisto, Andrea Ganna, Vincent Llorens, Hannele Laivuori, Taru Tukiainen, Mary Pat Reeve, Henrike Heyne, Nina Mars, Joel Rämö, Elmo Saarentaus, Hanna Ollila, Rodos Rodosthenous, Satu Strausz, Tuula Palotie, Kimmo Palin, Javier Garcia-Tabuenca, Harri Siirtola, Tuomo Kiiskinen, Jiwoo Lee, Kristin Tsuo, Amanda Elliott, Kati Kristiansson, Mikko Arvas, Kati Hyvärinen, Jarmo Ritari, Olli Carpén, Johannes Kettunen, Katri Pylkäs, Eeva Sliz, Minna Karjalainen, Tuomo Mantere, Eeva Kangasniemi, Sami Heikkinen, Arto Mannermaa, Eija Laakkonen, Nina Pitkänen, Samuel Lessard, Clément Chatelain, Perttu Terho, Sirpa Soini, Jukka Partanen, Eero Punkka, Raisa Serpi, Sanna Siltanen, Veli-Matti Kosma, Teijo Kuopio, Anu Jalanko, Huei-Yi Shen, Risto Kajanne, Mervi Aavikko, Henna Palin, Malla-Maria Linna, Mitja Kurki, Juha Karjalainen, Pietro Della Briotta Parolo, Arto Lehisto, Juha Mehtonen, Wei Zhou, Masahiro Kanai, Mutaamba Maasha, Hannele Laivuori, Aki Havulinna, Susanna Lemmelä, Tuomo Kiiskinen, L Elisa Lahtela, Mari Kaunisto, Elina Kilpeläinen, Timo P Sipilä, Oluwaseun Alexander Dada, Awaisa Ghazal, Anastasia Kytölä, Rigbe Weldatsadik, Sanni Ruotsalainen, Kati Donner, Timo P Sipilä, Anu Loukola, Päivi Laiho, Tuuli Sistonen, Essi Kaiharju, Markku Laukkanen, Elina Järvensivu, Sini Lähteenmäki, Lotta Männikkö, Regis Wong, Auli Toivola, Minna Brunfeldt, Hannele Mattsson, Kati Kristiansson, Susanna Lemmelä, Sami Koskelainen, Tero Hiekkalinna, Teemu Paajanen, Priit Palta, Kalle Pärn, Mart Kals, Shuang Luo, Tarja Laitinen, Mary Pat Reeve, Shanmukha Sampath Padmanabhuni, Marianna Niemi, Harri Siirtola, Javier Gracia-Tabuenca, Mika Helminen, Tiina Luukkaala, Iida Vähätalo, Jyrki Pitkänen, Marco Hautalahti, Johanna Mäkelä, Sarah Smith, Tom Southerington, Anni Kauko, Teemu Niiranen

**Affiliations:** Division of Perioperative Services, Intensive Care and Pain Medicine, Turku University Hospital, Turku, Finland; Department of Anesthesiology and Intensive Care, University of Turku, Turku, Finland; Division of Perioperative Services, Intensive Care and Pain Medicine, Turku University Hospital, Turku, Finland; Department of Anesthesiology and Intensive Care, University of Turku, Turku, Finland; Department of Internal Medicine, University of Turku, Turku, Finland; Department of Internal Medicine, University of Turku, Turku, Finland; Division of Medicine, Turku University Hospital, Turku, Finland; Department of Public Health Solutions, Finnish Institute for Health and Welfare, Turku, Finland

**Keywords:** Revascularization, Coronary artery bypass grafting, Percutaneous coronary intervention, Complication, Risk score, Genetic

## Abstract

**Figure:**
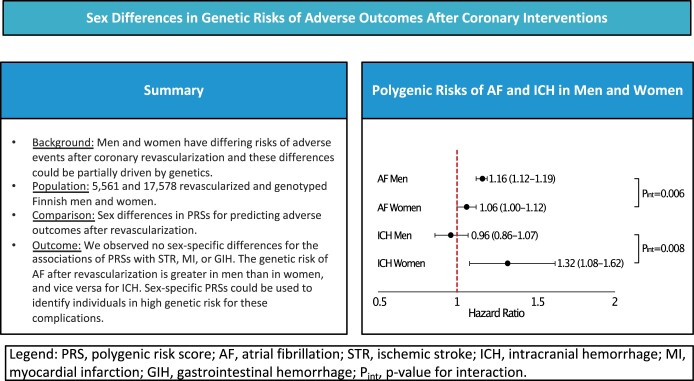

Men and women have differing risks of adverse events after revascularization procedures and these differences could be partially driven by genetics. We studied the sex-specific differences in associations of polygenic risk scores (PRSs) with atrial fibrillation (AF), ischaemic stroke (STR), intracranial haemorrhage (ICH), myocardial infarction (MI) and gastrointestinal haemorrhage (GIH) in coronary revascularization patients. The study cohort comprised 5561 and 17 578 revascularized women and men. All participants underwent genotyping and register-based follow-up from 1961 to 2021. We calculated PRSs for all individuals and used Cox models with interaction term to examine the sex-specific associations between the PRSs and adverse outcomes after revascularization. The AF-PRS was more strongly associated with AF in men [hazard ratio (HR) per 1 standard deviation increase, 1.16; 95% confidence interval (CI), 1.12–1.19; *P *=* *7.6 × 10^−22^) than in women (*P* for interaction 0.006). Conversely, ICH-PRS was more strongly associated with ICH after revascularization in women (HR, 1.32; 95% CI, 1.08–1.62; *P *=* *0.008) than in men (*P* for interaction 0.008). We observed no sex-specific differences for the associations of PRSs with STR, MI or GIH. The genetic risk of AF after revascularization is greater in men than in women, and vice versa for ICH. Sex-specific PRSs could be used to identify individuals in high genetic risk for these complications.

## INTRODUCTION

Coronary artery bypass grafting (CABG) and percutaneous coronary intervention (PCI) are related to adverse outcomes such as atrial fibrillation (AF), myocardial infarction (MI), ischaemic stroke (STR), intracranial haemorrhage (ICH) and gastrointestinal haemorrhage (GIH) [1]. Interestingly, sex-specific differences in risks of postprocedural complications have been observed [2].

The aetiology of sex differences in postprocedural complications is multifactorial extending from patient characteristics to perioperative care and the intensity and timing of the surgery itself. These differences could also be dependent on differences in genetic risk as polygenic risk scores (PRSs) have been previously shown to associate with certain adverse outcomes after revascularization [3].

To elucidate the role of genetics in sex differences in post-revascularization outcomes, we studied the association of sex- and disease-specific PRSs with long-term complications in 17 578 men and 5561 women who underwent coronary revascularization in Finland.

## MATERIALS AND METHODS

### Ethics statement

The approval of the Ethics Committee of the Hospital District of Helsinki and Uusimaa was obtained (HUS/990/2017). All participants provided informed written consent.

The study cohort consisted of 377 277 genotyped individuals from FinnGen data freeze 9 which included patients drawn from Finland’s national hospital biobanks and participants of Finnish cohort studies [4]. Of these, 23 139 individuals (5561 women, 17 578 men) had undergone a coronary revascularization procedure (PCI and/or CABG) and were considered for further analysis.

In total, 10 938 (1807 women) had undergone PCI and 14 723 (4195 women) CABG during the follow-up period from 1969 to October 11, 2021. If the patient had undergone both PCI and CABG, the first event was selected in the combined PCI/CABG group. When PCI and CABG groups were analysed separately, then only the first PCI or CABG event was selected for analysis.

The DNA samples were genotyped with Illumina and Affymetrix arrays. After quality control, the genotypes were imputed using a population-specific SISu v4.0 reference panel. Disease-specific PRSs were computed using the PRS-CS (continuous shrinkage) pipeline with default parameters. PRS-CS computes SNP effect sizes by high-dimensional Bayesian regression with CS priors using the obtained GWAS summary statistics and a linkage disequilibrium reference panel [4]. The summary statistics for calculating the PRSs were obtained from UK Biobank [3]. The European linkage disequilibrium reference panel was derived from samples of the 1000 Genomes Project. The PRSs were based on 1 098 015 genetic variants common in the linkage disequilibrium reference panel and FinnGen.

The selected end-point events for this study were AF, MI, STR, ICH and GIH and the corresponding International Classification of Diseases (ICD) codes used in this study are listed in Supplementary Material, Table S1 in the supplement. The genotyped study participants were linked to end-points using unique personal identification numbers and ICD codes derived from nationwide Hospital Discharge and Causes-of-Death registers. These diagnoses in the registers were made by the attending physician and the accuracy of the diagnoses and registers are robust and have been described in detail previously [5].

Separate datasets were derived for different revascularization procedures (PCI, CABG and PCI or CABG), for both sexes and for each outcome. Individuals with AF, STR, ICH, MI or GIH prior to revascularization were excluded from the analyses and thus the final study sample size and characteristics varied slightly depending on the outcome of interest (Table 1 and Supplementary Material, Table S2).

**Table 1:** Association between disease-specific polygenic risk score and adverse outcomes after revascularization procedures

PRS	Men			Women			Interaction
	HR (95% CI)	*P*-value	Cases/controls	HR (95% CI)	*P*-value	Cases/controls	
CABG/PCI							
AF	1.16 (1.12–1.19)	7.6 × 10^−22^	4543/10881	1.06 (1.00–1.12)	0.054	1172/3823	0.006
ICH	0.96 (0.86–1.07)	0.45	355/16958	1.32 (1.08–1.62)	0.008	94/5379	0.008
MI	1.02 (0.97–1.07)	0.34	1744/7717	1.06 (0.97–1.15)	0.18	511/2935	0.57
STR	1.04 (1.00–1.09)	0.07	2096/14370	1.09 (1.00–1.19)	0.05	539/4678	0.36
GIH	1.13 (1.06–1.21)	2.2 × 10^−4^	900/16314	1.08 (0.95–1.23)	0.25	230/5226	0.52
CABG							
AF	1.14 (1.10–1.18)	6.3 × 10^−12^	2876/5150	1.05 (0.95–1.12)	0.30	546/1057	0.11
ICH	0.95 (0.83–1.08)	0.44	219/8787	1.32 (0.99–1.75)	0.06	49/1733	0.07
MI	1.02 (0.95–1.08)	0.57	1032/43530	1.14 (1.01–1.30)	0.05	227/949	0.09
STR	1.06 (1.01–1.13)	0.03	1325/7229	1.09 (0.95–1.23)	0.21	250/1454	0.85
GIH	1.12 (1.03–1.23)	0.01	514/8456	1.14 (0.93–1.40)	0.21	92/1688	0.79
PCI							
AF	1.19 (1.14–1.24)	3.0 × 10^−15^	2228/6819	1.05 (0.99–1.14)	0.15	738/3002	0.003
ICH	0.99 (0.85–1.15)	0.89	171/10183	1.31 (1.03–1.75)	0.05	56/4064	0.07
MI	1.02 (0.95–1.09)	0.54	901/4019	1.05 (0.92–1.14)	0.37	335/2141	0.95
STR	1.00 (0.94–1.07)	0.99	1006/8798	1.06 (0.96–1.18)	0.26	346/3578	0.29
GIH	1.15 (1.06–1.26)	0.002	500/9769	1.03 (0.89–1.21)	0.75	160/3949	0.18

HRs were calculated for 1 SD increase in PRS. Hazard models were adjusted by age, operation type, sample collection year, with the presence of hypertension, obesity, diabetes, hypercholesterolaemia, chronic kidney disease, genotyping batch and the first 10 genetic principal components. *P*-values <0.05 were considered significant.

AF: atrial fibrillation; CABG: coronary artery bypass grafting; CI: confidence interval; GIH: gastrointestinal haemorrhage; HRs: hazard ratios; ICH: intracranial haemorrhage; MI: myocardial infarction; PCI: percutaneous coronary intervention; PRS: polygenic risk score; SD: standard deviation; STR: ischaemic stroke.

We used Cox proportional hazards models with sex–PRS interactions to examine the sex differences in genetic risk for adverse outcomes. We adjusted for sample collection year, genotyping batch, first 10 genetic principal components, the age at the time of the first revascularization and with the presence of hypertension, obesity, diabetes, hyperlipidemia and chronic kidney disease at baseline. The proportional hazards assumption was validated by visual inspection of log-minus-log plots due to the large sample size. Furthermore, we categorized the individuals in any revascularization group (PCI or CABG) into quartiles by their AF-PRS and ICH-PRS count.

In all analyses, we considered two-tailed *P*-values of 0.05 as statistically significant. We used R v.4.2.1 for all analyses.

## RESULTS

**Figure 1:**
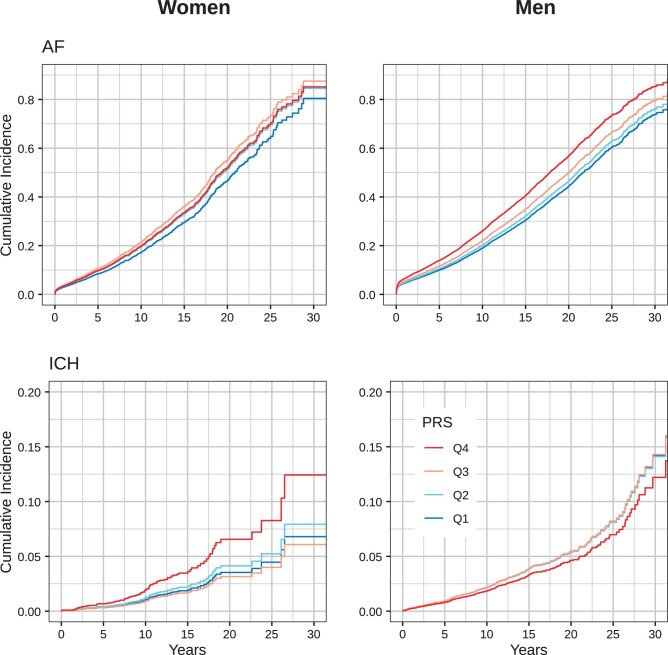
Cumulative incidence of AF and ICH after any revascularization procedure by AF-PRS and ICH-PRS quartiles. Q1: PRS <25th percentile; Q2: PRS in 25th to 50th percentile; Q3: PRS in 50th to 75th percentile; Q4: PRS >75th percentile. Models were adjusted by age, sample collection year, type of the first revascularization, genotyping batch, the first 10 genetic principal components, and with presence of hypertension, obesity, diabetes, hyperlipidemia, and chronic kidney disease at baseline. AF: atrial fibrillation; ICH: intracranial haemorrhage; PRS: polygenic risk score.

The study sample characteristics are reported in Supplementary Material, Table S2. The main results of the study are presented in Fig. 1 and Table 1. After CABG, AF-PRS, STR-PRS and GIH-PRS were associated to their respective outcomes in men, whereas after PCI, ICH-PRS was associated with ICH in women. In individuals with either type of revascularization, AF-PRS was associated with future AF more strongly in men than in women [hazard ratio (HR) 1.16 vs 1.06; *P* for interaction 0.006]. ICH-PRS was more strongly associated with future ICH in revascularized women than in men (HR 1.32 vs 0.96; *P* for interaction 0.008). These HRs were in general similar for CABG and PCI (Table 1). However, the sex interaction *P*-values were only borderline significant (*P* = 0.05–0.11) due to lack of statistical power, except for AF-PRS post-PCI (*P* = 0.003).

## DISCUSSION

In this study of 23 139 revascularized men and women, we demonstrate sex differences in the genetic risk for post-revascularization outcomes. Namely, AF-PRS is more strongly associated with future AF in men and ICH-PRS is more closely related to incident ICH in women. We observed no sex differences of sex-specific PRSs with MI, STR and GIH.

AF is common after revascularization with a prevalence rate of 15–40% and 12% after CABG and PCI, respectively [1, 6]. It is also known that AF is more common in men [7]. The heritability and genetic contribution for AF in the general population have been confirmed in several studies [8]. However, research on the influence of genetics on sex-specific risk differences has yielded mixed and rather modest results [7]. Our results suggest that the genetic susceptibility for AF differs in men and women, either directly or through mediating factors. Therefore, as postoperative AF is associated with increased mortality, morbidity and cost after revascularization, genetic profiling before operative treatment with disease-specific PRSs could help identify individuals at high genetic risk and guide them to appropriate follow-up and therapy.

We also observed a stronger genetic susceptibility for ICH in revascularized women than in men. In women, ICH-PRS was associated with 32–34% greater risk for ICH than in men, depending on the type of revascularization. In prior studies, PRSs have been associated with ischaemic stroke in the general population but the data on possible sex-specific differences on the association between ICH-PRS and ICH are more limited [9]. Our current data suggest that the genetic risk for ICH in the post-revascularization setting is greater in women than in men. Further, as ICH is a devastating complication, genetic profiling and identification of individuals with high ICH-PRS could help individualize postoperative antithrombotic and antihypertensive therapy.

In contrast to previous studies [9], we observed no association between STR-PRS and stroke, and no association was observed between MI-PRS and MI. This may be due to the strong, independent effects of PCI, CABG and the aggressive drug therapy related to them, which may have diminished the links of genetic factors with stroke and MI.

Genetic polymorphisms have been previously associated with increased risk for upper gastrointestinal bleeding due to adverse drug reactions from antithrombotic and anticoagulant medications [10]. We observed that men with revascularization had increased genetic risk for GIH but this association was non-significant in women, possibly due to the lower number of women in the study sample. However, this difference was non-significant.

### Limitations

Finally, these results must be weighed against some limitations. First, exclusion of prevalent cases may cause individuals with the greatest genetic risk to be excluded. Second, as CVD is more common in men, only 24% of the study participants were women, resulting in a lack of statistical power. Third, given that the sample comprised mainly of individuals of Northern European ancestry, our results may not be generalizable to other populations. Fourth, other acquired clinical risk factors for AF and ICH were not available in this study.

## CONCLUSION

Based on our findings, the genetic risk for complications after revascularization is sex specific. The genetic risk of AF after revascularization is significantly higher in men. Women, on the other hand, carry an increased genetic risk for ICH after these procedures. Additional research is warranted to elucidate the underlying mechanisms in these genetic sex differences and to evaluate the possible potential of PRS screening and pretreatment of these high-risk patients.

## Supplementary Material

ivae006_Supplementary_Data

## Data Availability

Any researcher can apply for the register data from the Finnish Data Authority and for individual-level genotype data from Finnish biobanks via the Fingenious portal. Data analysis and processing pipelines used are available at: https://github.com/akauko/cabg_prs_sex.
