# Supplementary material for: Sex-specific genetic risks for adverse outcomes after coronary revascularization procedures
Source: Interdiscip Cardiovasc Thorac Surg. 2024 Jan 12;38(1):ivae006. doi: 10.1093/icvts/ivae006 (PMC10799751; doi:10.1093/icvts/ivae006)
Supplement: ivae006_Supplementary_Data [file ivae006_supplementary_data.docx]

**Supplemental Material**

**Supplemental Table 1.** Endpoint definitions in FinnGen and in national registers.

| Endpoint | FinnGen code | Hospital discharge and death registries | Drug reimbursement and purchase registries |
| --- | --- | --- | --- |
| Revascularization^1^ | I9_REVASC | I9_CABG or I9_ANGIO |  |
| CABG^1^ | I9_CABG | NOMESCO: FNA\|FNB\|FNC\|FND\|FNE, HL: 531[1-5]  HP1: 11\|25\|111\|112\|113\|119, HP2: AA1\|AA2\|AA3\|AAX |  |
| PCI^1^ | I9_ANGIO | NOMESCO: FNF\|FNG\|TFN40\|FN1AT\|FN1BT\|FN1YT HP1: 82\|83\|84, HP2: AN2\|AN3\|AN4\|ANA\|ANB |  |
| AF | I9_AF | ICD-10: I48, ICD-9: 4273, ICD-8: 42792 | DR:207\|351\|342, ICD-10:I48 |
| MI | I9_MI | ICD-10: I21\|I22, ICD-9: 410, ICD-8: 410 |  |
| Stroke | I9_STR | ICD-10: I61\|I63\|I64 (excluding I636), ICD-9: 431\|4330A\| 4331A\|4339A\|4340A\|4341A\|4349A\|436, ICD-8: 431\|433\| 434\|436 (excluding 43101\|43191) |  |
| ICH | I9_INTRACRA | ICD-10: I60\|I61, ICD-9: 430\|431, ICD-8: 430\|431 |  |
| GIH | K11_GIBLEEDING | ICD-10: K92[0-2], ICD-9: 578 |  |
| Hypertension | I9_HYPTENS | ICD-10: I10-I15, I67.4, ICD-9: 4019X\|4029A\|4029B\| 4039A\|4040A\|4059A\|4059B\|4372A\|4059X, ICD-8: 400\|401\|402\|403\|404 | DR: 205 |
| Obesity | E4_OBESITY | ICD-10: E66, ICD-9: 2780\|2788A, ICD-8: 277.99 |  |
| Diabetes | E4_DIABETES | ICD-10: E10, E11, E12 | ATC: A10B |
| Hypercholesterolemia | E4_HYPERCHOL, RX_STATIN | ICD-10: E78, ICD-9: 2720, ICD-8: 2720 | ATC: C10AA |
| Chronic Kidney Disease | N14_CHRONKIDNEYDIS | ICD-10: N18, ICD-9: 585 | DR: 138 |

The endpoints of this study were obtained from Hospital Discharge, Cause of Death, Drug reimbursement and Drug purchase registries. Diseases in the registers were defined by ICD-codes and Finnish Social Insurance Institution drug codes (DR: Drug reimbursement code, ATC: Anatomical therapeutic chemical code). Operations were defined using Nordic NOMESCO codes and national codes (HL: Finnish Hospital League procedure codes; HP1 and HP2: Demanding heart patient procedure codes). CABG; Coronary artery bypass grafting, PCI; percutaneous coronary intervention AF; atrial fibrillation, MI; myocardial infarction, ICH; intracranial hemorrhage, GIH; gastrointestinal hemorrhage. ^1^Not available in death registry.

**Supplemental Table 2.** Characteristics of the combined revascularization group.

|  | Women | | | | Men | | | |
| --- | --- | --- | --- | --- | --- | --- | --- | --- |
| Characteristics | AF | No AF | ICH | No ICH | AF | No AF | ICH | No ICH |
| Number of Patients | 1172 | 3823 | 94 | 5379 | 4543 | 10881 | 355 | 16958 |
| Follow up, years (SD) | 7.9 (6.5) | 9.1 (6.5) | 9.4 (6.0) | 9.8 (6.7) | 8.5 (7.2) | 11.1 (7.1) | 9.5 (7.2) | 11.7 (7.4) |
| Age, years (SD) | 68.0 (9.5) | 64.4 (12.1) | 66.9 (9.4) | 66.0 (11.5) | 64.0 (9.5) | 63.0 (10.4) | 64.5 (9.6) | 64.1 (10.2) (9.4) |
| CABG, n (%) | 487 (41.6) | 916 (24) | 42 (44.7) | 1501 (27.9) | 2609 (57.4) | 4588 (42.2) | 207 (58.3) | 7812 (46.1) |
| Hypertension, n (%) | 642 (54.8) | 1712 (44.8) | 49 (52.1) | 2633 (48.9) | 1995 (43.9) | 4117 (37.8) | 159 (44.8) | 7024 (41.4) |
| Obesity, n (%) | 42 (3.6) | 159 (4.2) | <5 | 223 (4.1) | 112 (2.5) | 241 (2.2) | 7 (2.0) | 450 (2.7) |
| Diabetes, n (%) | 282 (24.1) | 980 (25.6) | 25 (26.6) | 1374 (25.5) | 904 (19.9) | 2380 (21.9) | 76 (21.4) | 3756 (22.1) |
| Hypercholesterolemia, n (%) | 725 (61.9) | 2174 (56.9) | 46 (48.9) | 3174 (59.0) | 2354 (51.8) | 6093 (56.0) | 188 (53.0) | 9525 (56.2) |
| Chronic kidney disease, n (%) | 13 (1.1) | 59 (1.5) | <5 | 79 (1.5) | 50 (1.1) | 176 (1.6) | 7 (2.0) | 272 (1.6) |

Prevalent cases before PCI or CABG procedures were excluded. CABG, coronary artery bypass grafting; PCI, percutaneous coronary intervention; AF, atrial fibrillation; ICH, Intracranial hemorrhage; SD, standard deviation. Characteristics with n < 5 are not shown due to privacy policy of the FinnGen study.

**FinnGen Collaborators**

| **Full Name** | **Affiliation** | **E-mail** | **Role 1** | **Role 2** |
| --- | --- | --- | --- | --- |
| Aarno Palotie | Institute for Molecular Medicine Finland (FIMM), HiLIFE, University of Helsinki, Helsinki, Finland; Broad Institute of MIT and Harvard; Massachusetts General Hospital | aarno.palotie@helsinki.fi | Steering Committee | Steering Committee |
| Mark Daly | Institute for Molecular Medicine Finland (FIMM), HiLIFE, University of Helsinki, Helsinki, Finland; Broad Institute of MIT and Harvard; Massachusetts General Hospital | mark.daly@helsinki.fi | Steering Committee | Steering Committee |
| Bridget Riley-Gills | Abbvie, Chicago, IL, United States | bridget.rileygillis@abbvie.com | Steering Committee | Pharmaceutical companies |
| Howard Jacob | Abbvie, Chicago, IL, United States | howard.jacob@abbvie.com | Steering Committee | Pharmaceutical companies |
| Dirk Paul | Astra Zeneca, Cambridge, United Kingdom | dirk.paul@astrazeneca.com | Steering Committee | Pharmaceutical companies |
| Athena Matakidou | Astra Zeneca, Cambridge, United Kingdom | athena.x.matakidou@gsk.com | Steering Committee | Pharmaceutical companies |
| Adam Platt | Astra Zeneca, Cambridge, United Kingdom | adam.platt@astrazeneca.com | Steering Committee | Pharmaceutical companies |
| Heiko Runz | Biogen, Cambridge, MA, United States | heiko.runz@biogen.com | Steering Committee | Pharmaceutical companies |
| Sally John | Biogen, Cambridge, MA, United States | sally.john@biogen.com | Steering Committee | Pharmaceutical companies |
| George Okafo | Boehringer Ingelheim, Ingelheim am Rhein, Germany | george.okafo@boehringer-ingelheim.com | Steering Committee | Pharmaceutical companies |
| Nathan Lawless | Boehringer Ingelheim, Ingelheim am Rhein, Germany | nathan.lawless@boehringer-ingelheim.com | Steering Committee | Pharmaceutical companies |
| Heli Salminen-Mankonen | Boehringer Ingelheim, Ingelheim am Rhein, Germany | heli.salminen-mankonen@boehringer-ingelheim.com | Steering Committee | Pharmaceutical companies |
| Robert Plenge | Bristol Myers Squibb, New York, NY, United States | robert.plenge@bms.com | Steering Committee | Pharmaceutical companies |
| Joseph Maranville | Bristol Myers Squibb, New York, NY, United States | joseph.maranville@bms.com | Steering Committee | Pharmaceutical companies |
| Mark McCarthy | Genentech, San Francisco, CA, United States | mccarthy.mark@gene.com | Steering Committee | Pharmaceutical companies |
| Julie Hunkapiller | Genentech, San Francisco, CA, United States | hunkapiller.julie@gene.com | Steering Committee | Pharmaceutical companies |
| Margaret G. Ehm | GlaxoSmithKline, Collegeville, PA, United States | meg.g.ehm@gsk.com | Steering Committee | Pharmaceutical companies |
| Kirsi Auro | GlaxoSmithKline, Espoo, Finland | kirsi.m.auro@gsk.com | Steering Committee | Pharmaceutical companies |
| Simonne Longerich | Merck, Kenilworth, NJ, United States | simonne.longerich@merck.com | Steering Committee | Pharmaceutical companies |
| Caroline Fox | Merck, Kenilworth, NJ, United States | caroline.fox@merck.com | Steering Committee | Pharmaceutical companies |
| Anders Mälarstig | Pfizer, New York, NY, United States | anders.malarstig@pfizer.com | Steering Committee | Pharmaceutical companies |
| Katherine Klinger | Translational Sciences, Sanofi R&D, Framingham, MA, USA | katherine.klinger@sanofi.com | Steering Committee | Pharmaceutical companies |
| Deepak Raipal | Translational Sciences, Sanofi R&D, Framingham, MA, USA | deepak.rajpal@sanofi.com | Steering Committee | Pharmaceutical companies |
| Eric Green | Maze Therapeutics, San Francisco, CA, United States | egreen@mazetx.com | Steering Committee | Pharmaceutical companies |
| Robert Graham | Maze Therapeutics, San Francisco, CA, United States | rgraham@mazetx.com | Steering Committee | Pharmaceutical companies |
| Robert Yang | Janssen Biotech, Beerse, Belgium | ryang31@its.jnj.com | Steering Committee | Pharmaceutical companies |
| Chris O´Donnell | Novartis Institutes for BioMedical Research, Cambridge, MA, United States | chris.odonnell@novartis.com | Steering Committee | Pharmaceutical companies |
| Tomi P. Mäkelä | HiLIFE, University of Helsinki, Finland, Finland | tomi.makela@helsinki.fi | Steering Committee | University of Helsinki & Biobanks |
| Jaakko Kaprio | Institute for Molecular Medicine Finland (FIMM), HiLIFE, University of Helsinki, Helsinki, Finland | jaakko.kaprio@helsinki.fi | Steering Committee | University of Helsinki & Biobanks |
| Petri Virolainen | Auria Biobank / University of Turku / Hospital District of Southwest Finland, Turku, Finland | petri.virolainen@tyks.fi | Steering Committee | University of Helsinki & Biobanks |
| Antti Hakanen | Auria Biobank / University of Turku / Hospital District of Southwest Finland, Turku, Finland | antti.hakanen@tyks.fi | Steering Committee | University of Helsinki & Biobanks |
| Terhi Kilpi | THL Biobank / Finnish Institute for Health and Welfare (THL), Helsinki, Finland | terhi.kilpi@thl.fi | Steering Committee | University of Helsinki & Biobanks |
| Markus Perola | THL Biobank / Finnish Institute for Health and Welfare (THL), Helsinki, Finland | markus.perola@thl.fi | Steering Committee | University of Helsinki & Biobanks |
| Jukka Partanen | Finnish Red Cross Blood Service / Finnish Hematology Registry and Clinical Biobank, Helsinki, Finland | jukka.partanen@veripalvelu.fi | Steering Committee | University of Helsinki & Biobanks |
| Anne Pitkäranta | Helsinki Biobank / Helsinki University and Hospital District of Helsinki and Uusimaa, Helsinki | anne.pitkaranta@hus.fi | Steering Committee | University of Helsinki & Biobanks |
| Taneli Raivio | Helsinki Biobank / Helsinki University and Hospital District of Helsinki and Uusimaa, Helsinki | taneli.raivio@hus.fi | Steering Committee | University of Helsinki & Biobanks |
| Raisa Serpi | Northern Finland Biobank Borealis / University of Oulu / Northern Ostrobothnia Hospital District, Oulu, Finland | raisa.serpi@ppshp.fi | Steering Committee | University of Helsinki & Biobanks |
| Tarja Laitinen | Finnish Clinical Biobank Tampere / University of Tampere / Pirkanmaa Hospital District, Tampere, Finland | tarja.laitinen@pshp.fi | Steering Committee | University of Helsinki & Biobanks |
| Veli-Matti Kosma | Biobank of Eastern Finland / University of Eastern Finland / Northern Savo Hospital District, Kuopio, Finland | veli-matti.kosma@uef.fi | Steering Committee | University of Helsinki & Biobanks |
| Jari Laukkanen | Central Finland Biobank / University of Jyväskylä / Central Finland Health Care District, Jyväskylä, Finland | jari.laukkanen@ksshp.fi | Steering Committee | University of Helsinki & Biobanks |
| Marco Hautalahti | FINBB - Finnish biobank cooperative | marco.hautalahti@finbb.fi | Steering Committee | University of Helsinki & Biobanks |
| Outi Tuovila | Business Finland, Helsinki, Finland | outi.tuovila@businessfinland.fi | Steering Committee | Other Experts/ Non-Voting Members |
| Raimo Pakkanen | Business Finland, Helsinki, Finland | raimo.pakkanen@businessfinland.fi | Steering Committee | Other Experts/ Non-Voting Members |
| Jeffrey Waring | Abbvie, Chicago, IL, United States | jeff.waring@abbvie.com | Scientific Committee | Pharmaceutical companies |
| Bridget Riley-Gillis | Abbvie, Chicago, IL, United States | bridget.rileygillis@abbvie.com | Scientific Committee | Pharmaceutical companies |
| Fedik Rahimov | Abbvie, Chicago, IL, United States | fedik.rahimov@abbvie.com | Scientific Committee | Pharmaceutical companies |
| Ioanna Tachmazidou | Astra Zeneca, Cambridge, United Kingdom | ioanna.tachmazidou@astrazeneca.com | Scientific Committee | Pharmaceutical companies |
| Chia-Yen Chen | Biogen, Cambridge, MA, United States | chiayen.chen@biogen.com | Scientific Committee | Pharmaceutical companies |
| Heiko Runz | Biogen, Cambridge, MA, United States | heiko.runz@biogen.com | Scientific Committee | Pharmaceutical companies |
| Zhihao Ding | Boehringer Ingelheim, Ingelheim am Rhein, Germany | zhihao.ding@boehringer-ingelheim.com | Scientific Committee | Pharmaceutical companies |
| Marc Jung | Boehringer Ingelheim, Ingelheim am Rhein, Germany | marc_oliver.jung@boehringer-ingelheim.com | Scientific Committee | Pharmaceutical companies |
| Shameek Biswas | Bristol Myers Squibb, New York, NY, United States | Shameek.Biswas@bms.com | Scientific Committee | Pharmaceutical companies |
| Rion Pendergrass | Genentech, San Francisco, CA, United States | penders2@gene.com | Scientific Committee | Pharmaceutical companies |
| Julie Hunkapiller | Genentech, San Francisco, CA, United States | hunkapiller.julie@gene.com | Scientific Committee | Pharmaceutical companies |
| Margaret G. Ehm | GlaxoSmithKline, Collegeville, PA, United States | meg.g.ehm@gsk.com | Scientific Committee | Pharmaceutical companies |
| David Pulford | GlaxoSmithKline, Stevenage, United Kingdom | david.x.pulford@gsk.com | Scientific Committee | Pharmaceutical companies |
| Neha Raghavan | Merck, Kenilworth, NJ, United States | neha.raghavan@merck.com | Scientific Committee | Pharmaceutical companies |
| Adriana Huertas-Vazquez | Merck, Kenilworth, NJ, United States | adriana.huertas.vazquez@merck.com | Scientific Committee | Pharmaceutical companies |
| Jae-Hoon Sul | Merck, Kenilworth, NJ, United States | jae.hoon.sul@merck.com | Scientific Committee | Pharmaceutical companies |
| Anders Mälarstig | Pfizer, New York, NY, United States | anders.malarstig@pfizer.com | Scientific Committee | Pharmaceutical companies |
| Xinli Hu | Pfizer, New York, NY, United States | xinli.hu@pfizer.com | Scientific Committee | Pharmaceutical companies |
| Katherine Klinger | Translational Sciences, Sanofi R&D, Framingham, MA, USA | katherine.klinger@sanofi.com | Scientific Committee | Pharmaceutical companies |
| Robert Graham | Maze Therapeutics, San Francisco, CA, United States | rgraham@mazetx.com | Scientific Committee | Pharmaceutical companies |
| Eric Green | Maze Therapeutics, San Francisco, CA, United States | egreen@mazetx.com | Scientific Committee | Pharmaceutical companies |
| Sahar Mozaffari | Maze Therapeutics, San Francisco, CA, United States | smozaffari@mazetx.com | Scientific Committee | Pharmaceutical companies |
| Dawn Waterworth | Janssen Research & Development, LLC, Spring House, PA, United States | dwaterwo@its.jnj.com | Scientific Committee | Pharmaceutical companies |
| Nicole Renaud | Novartis Institutes for BioMedical Research, Cambridge, MA, United States | nicole.renaud@novartis.com | Scientific Committee | Pharmaceutical companies |
| Ma´en Obeidat | Novartis Institutes for BioMedical Research, Cambridge, MA, United States | maen.obeidat@novartis.com | Scientific Committee | Pharmaceutical companies |
| Samuli Ripatti | Institute for Molecular Medicine Finland (FIMM), HiLIFE, University of Helsinki, Helsinki, Finland | samuli.ripatti@helsinki.fi | Scientific Committee | University of Helsinki & Biobanks |
| Johanna Schleutker | Auria Biobank / Univ. of Turku / Hospital District of Southwest Finland, Turku, Finland | johanna.schleutker@utu.fi | Scientific Committee | University of Helsinki & Biobanks |
| Markus Perola | THL Biobank / Finnish Institute for Health and Welfare (THL), Helsinki, Finland | markus.perola@thl.fi | Scientific Committee | University of Helsinki & Biobanks |
| Mikko Arvas | Finnish Red Cross Blood Service / Finnish Hematology Registry and Clinical Biobank, Helsinki, Finland | mikko.arvas@veripalvelu.fi | Scientific Committee | University of Helsinki & Biobanks |
| Olli Carpén | Helsinki Biobank / Helsinki University and Hospital District of Helsinki and Uusimaa, Helsinki | olli.carpen@helsinki.fi | Scientific Committee | University of Helsinki & Biobanks |
| Reetta Hinttala | Northern Finland Biobank Borealis / University of Oulu / Northern Ostrobothnia Hospital District, Oulu, Finland | reetta.hinttala@oulu.fi | Scientific Committee | University of Helsinki & Biobanks |
| Johannes Kettunen | Northern Finland Biobank Borealis / University of Oulu / Northern Ostrobothnia Hospital District, Oulu, Finland | johannes.kettunen@oulu.fi | Scientific Committee | University of Helsinki & Biobanks |
| Arto Mannermaa | Biobank of Eastern Finland / University of Eastern Finland / Northern Savo Hospital District, Kuopio, Finland | arto.mannermaa@uef.fi | Scientific Committee | University of Helsinki & Biobanks |
| Katriina Aalto-Setälä | Faculty of Medicine and Health Technology, Tampere University, Tampere, Finland | katriina.aalto-setala@tuni.fi | Scientific Committee | University of Helsinki & Biobanks |
| Mika Kähönen | Finnish Clinical Biobank Tampere / University of Tampere / Pirkanmaa Hospital District, Tampere, Finland | mika.kahonen@uta.fi | Scientific Committee | University of Helsinki & Biobanks |
| Jari Laukkanen | Central Finland Biobank / University of Jyväskylä / Central Finland Health Care District, Jyväskylä, Finland | jari.laukkanen@ksshp.fi | Scientific Committee | University of Helsinki & Biobanks |
| Johanna Mäkelä | FINBB - Finnish biobank cooperative | johanna.makela@finbb.fi | Scientific Committee | University of Helsinki & Biobanks |
| Reetta Kälviäinen | Northern Savo Hospital District, Kuopio, Finland | reetta.kalviainen@kuh.fi | Clinical Groups | Neurology Group |
| Valtteri Julkunen | Northern Savo Hospital District, Kuopio, Finland | valtteri.julkunen@kuh.fi | Clinical Groups | Neurology Group |
| Hilkka Soininen | Northern Savo Hospital District, Kuopio, Finland | hilkka.soininen@uef.fi | Clinical Groups | Neurology Group |
| Anne Remes | Northern Ostrobothnia Hospital District, Oulu, Finland | anne.remes@oulu.fi | Clinical Groups | Neurology Group |
| Mikko Hiltunen | University of Eastern Finland, Kuopio, Finland | mikko.hiltunen@uef.fi | Clinical Groups | Neurology Group |
| Jukka Peltola | Pirkanmaa Hospital District, Tampere, Finland | jukka.peltola@pshp.fi | Clinical Groups | Neurology Group |
| Minna Raivio | Hospital District of Helsinki and Uusimaa, Helsinki, Finland | minna.raivio@geri.fi | Clinical Groups | Neurology Group |
| Pentti Tienari | Hospital District of Helsinki and Uusimaa, Helsinki, Finland | pentti.tienari@hus.fi | Clinical Groups | Neurology Group |
| Juha Rinne | Hospital District of Southwest Finland, Turku, Finland | juha.rinne@tyks.fi | Clinical Groups | Neurology Group |
| Roosa Kallionpää | Hospital District of Southwest Finland, Turku, Finland | roosa.kallionpaa@tyks.fi | Clinical Groups | Neurology Group |
| Juulia Partanen | Institute for Molecular Medicine Finland, HiLIFE, University of Helsinki, Finland | juulia.partanen@helsinki.fi | Clinical Groups | Neurology Group |
| Ali Abbasi | Abbvie, Chicago, IL, United States | ali.abbasi@abbvie.com | Clinical Groups | Neurology Group |
| Adam Ziemann | Abbvie, Chicago, IL, United States | adam.ziemann@abbvie.com | Clinical Groups | Neurology Group |
| Nizar Smaoui | Abbvie, Chicago, IL, United States | nizar.smaoui@abbvie.com | Clinical Groups | Neurology Group |
| Anne Lehtonen | Abbvie, Chicago, IL, United States | anne.lehtonen@abbvie.com | Clinical Groups | Neurology Group |
| Susan Eaton | Biogen, Cambridge, MA, United States | susan.eaton@biogen.com | Clinical Groups | Neurology Group |
| Heiko Runz | Biogen, Cambridge, MA, United States | heiko.runz@biogen.com | Clinical Groups | Neurology Group |
| Sanni Lahdenperä | Biogen, Cambridge, MA, United States | sanni.lahdenpera@biogen.com | Clinical Groups | Neurology Group |
| Shameek Biswas | Bristol Myers Squibb, New York, NY, United States | shameek.biswas@bms.com | Clinical Groups | Neurology Group |
| Julie Hunkapiller | Genentech, San Francisco, CA, United States | hunkapiller.julie@gene.com | Clinical Groups | Neurology Group |
| Natalie Bowers | Genentech, San Francisco, CA, United States | bowersn1@gene.com | Clinical Groups | Neurology Group |
| Edmond Teng | Genentech, San Francisco, CA, United States | teng.edmond@gene.com | Clinical Groups | Neurology Group |
| Rion Pendergrass | Genentech, San Francisco, CA, United States | penders2@gene.com | Clinical Groups | Neurology Group |
| Fanli Xu | GlaxoSmithKline, Brentford, United Kingdom | chun-fang.2.xu@gsk.com | Clinical Groups | Neurology Group |
| David Pulford | GlaxoSmithKline, Stevenage, United Kingdom | david.x.pulford@gsk.com | Clinical Groups | Neurology Group |
| Kirsi Auro | GlaxoSmithKline, Espoo, Finland | kirsi.m.auro@gsk.com | Clinical Groups | Neurology Group |
| Laura Addis | GlaxoSmithKline, Brentford, United Kingdom | laura.x.addis@gsk.com | Clinical Groups | Neurology Group |
| John Eicher | GlaxoSmithKline, Brentford, United Kingdom | john.d.eicher@gsk.com | Clinical Groups | Neurology Group |
| Qingqin S Li | Janssen Research & Development, LLC, Titusville, NJ 08560, United States | QLi2@its.jnj.com | Clinical Groups | Neurology Group |
| Karen He | Janssen Research & Development, LLC, Spring House, PA, United States | khe2@its.jnj.com | Clinical Groups | Neurology Group |
| Ekaterina Khramtsova | Janssen Research & Development, LLC, Spring House, PA, United States | ekhramts@its.jnj.com | Clinical Groups | Neurology Group |
| Neha Raghavan | Merck, Kenilworth, NJ, United States | neha.raghavan@merck.com | Clinical Groups | Neurology Group |
| Martti Färkkilä | Hospital District of Helsinki and Uusimaa, Helsinki, Finland | martti.farkkila@hus.fi | Clinical Groups | Gastroenterology Group |
| Jukka Koskela | Hospital District of Helsinki and Uusimaa, Helsinki, Finland | jukka.koskela@helsinki.fi | Clinical Groups | Gastroenterology Group |
| Sampsa Pikkarainen | Hospital District of Helsinki and Uusimaa, Helsinki, Finland | sampsa.pikkarainen@hus.fi | Clinical Groups | Gastroenterology Group |
| Airi Jussila | Pirkanmaa Hospital District, Tampere, Finland | airi.jussila@pshp.fi | Clinical Groups | Gastroenterology Group |
| Katri Kaukinen | Pirkanmaa Hospital District, Tampere, Finland | katri.kaukinen@tuni.fi | Clinical Groups | Gastroenterology Group |
| Timo Blomster | Northern Ostrobothnia Hospital District, Oulu, Finland | timo.blomster@ppshp.fi | Clinical Groups | Gastroenterology Group |
| Mikko Kiviniemi | Northern Savo Hospital District, Kuopio, Finland | mikko.kiviniemi@kuh.fi | Clinical Groups | Gastroenterology Group |
| Markku Voutilainen | Hospital District of Southwest Finland, Turku, Finland | markku.voutilainen@tyks.fi | Clinical Groups | Gastroenterology Group |
| Mark Daly | Institute for Molecular Medicine, Finland (FIMM), HiLIFE, University of Helsinki, Helsinki, Finland; Broad Institute of MIT and Harvard; Massachusetts General Hospital | mark.daly@helsinki.fi | Clinical Groups | Gastroenterology Group |
| Ali Abbasi | Abbvie, Chicago, IL, United States | ali.abbasi@abbvie.com | Clinical Groups | Gastroenterology Group |
| Jeffrey Waring | Abbvie, Chicago, IL, United States | jeff.waring@abbvie.com | Clinical Groups | Gastroenterology Group |
| Nizar Smaoui | Abbvie, Chicago, IL, United States | nizar.smaoui@abbvie.com | Clinical Groups | Gastroenterology Group |
| Fedik Rahimov | Abbvie, Chicago, IL, United States | fedik.rahimov@abbvie.com | Clinical Groups | Gastroenterology Group |
| Anne Lehtonen | Abbvie, Chicago, IL, United States | anne.lehtonen@abbvie.com | Clinical Groups | Gastroenterology Group |
| Tim Lu | Genentech, San Francisco, CA, United States | lut8@gene.com | Clinical Groups | Gastroenterology Group |
| Natalie Bowers | Genentech, San Francisco, CA, United States | bowersn1@gene.com | Clinical Groups | Gastroenterology Group |
| Rion Pendergrass | Genentech, San Francisco, CA, United States | penders2@gene.com | Clinical Groups | Gastroenterology Group |
| Linda McCarthy | GlaxoSmithKline, Brentford, United Kingdom | linda.c.mccarthy@gsk.com | Clinical Groups | Gastroenterology Group |
| Amy Hart | Janssen Research & Development, LLC, Spring House, PA, United States | ahart13@its.jnj.com | Clinical Groups | Gastroenterology Group |
| Meijian Guan | Janssen Research & Development, LLC, Spring House, PA, United States | mguan4@its.jnj.com | Clinical Groups | Gastroenterology Group |
| Jason Miller | Merck, Kenilworth, NJ, United States | jason.miller4@merck.com | Clinical Groups | Gastroenterology Group |
| Kirsi Kalpala | Pfizer, New York, NY, United States | kirsi.kalpala@pfizer.com | Clinical Groups | Gastroenterology Group |
| Melissa Miller | Pfizer, New York, NY, United States | melissa.r.miller@pfizer.com | Clinical Groups | Gastroenterology Group |
| Xinli Hu | Pfizer, New York, NY, United States | xinli.hu@pfizer.com | Clinical Groups | Gastroenterology Group |
| Kari Eklund | Hospital District of Helsinki and Uusimaa, Helsinki, Finland | kari.eklund@hus.fi | Clinical Groups | Rheumatology Group |
| Antti Palomäki | Hospital District of Southwest Finland, Turku, Finland | ajpalo@utu.fi | Clinical Groups | Rheumatology Group |
| Pia Isomäki | Pirkanmaa Hospital District, Tampere, Finland | pia.isomaki@pshp.fi | Clinical Groups | Rheumatology Group |
| Laura Pirilä | Hospital District of Southwest Finland, Turku, Finland | laura.pirila@fimnet.fi,laura.pirila@tyks.fi | Clinical Groups | Rheumatology Group |
| Oili Kaipiainen-Seppänen | Northern Savo Hospital District, Kuopio, Finland | oili.kaipiainen-seppanen@kuh.fi | Clinical Groups | Rheumatology Group |
| Johanna Huhtakangas | Northern Ostrobothnia Hospital District, Oulu, Finland | johanna.huhtakangas@kuh.fi | Clinical Groups | Rheumatology Group |
| Nina Mars | Institute for Molecular Medicine Finland (FIMM), HiLIFE, University of Helsinki, Helsinki, Finland | nina.mars@helsinki.fi | Clinical Groups | Rheumatology Group |
| Ali Abbasi | Abbvie, Chicago, IL, United States | ali.abbasi@abbvie.com | Clinical Groups | Rheumatology Group |
| Jeffrey Waring | Abbvie, Chicago, IL, United States | jeff.waring@abbvie.com | Clinical Groups | Rheumatology Group |
| Fedik Rahimov | Abbvie, Chicago, IL, United States | fedik.rahimov@abbvie.com | Clinical Groups | Rheumatology Group |
| Apinya Lertratanakul | Abbvie, Chicago, IL, United States | apinya.lertratanakul@abbvie.com | Clinical Groups | Rheumatology Group |
| Nizar Smaoui | Abbvie, Chicago, IL, United States | nizar.smaoui@abbvie.com | Clinical Groups | Rheumatology Group |
| Anne Lehtonen | Abbvie, Chicago, IL, United States | anne.lehtonen@abbvie.com | Clinical Groups | Rheumatology Group |
| Marla Hochfeld | Bristol Myers Squibb, New York, NY, United States | mhochfeld@celgene.com | Clinical Groups | Rheumatology Group |
| Natalie Bowers | Genentech, San Francisco, CA, United States | bowersn1@gene.com | Clinical Groups | Rheumatology Group |
| Rion Pendergrass | Genentech, San Francisco, CA, United States | penders2@gene.com | Clinical Groups | Rheumatology Group |
| Jorge Esparza Gordillo | GlaxoSmithKline, Brentford, United Kingdom | jorge.x.esparza-gordillo@gsk.com | Clinical Groups | Rheumatology Group |
| Kirsi Auro | GlaxoSmithKline, Espoo, Finland | kirsi.m.auro@gsk.com | Clinical Groups | Rheumatology Group |
| Dawn Waterworth | Janssen Research & Development, LLC, Spring House, PA, United States | dwaterwo@its.jnj.com | Clinical Groups | Rheumatology Group |
| Fabiana Farias | Merck, Kenilworth, NJ, United States | fabiana.farias@merck.com | Clinical Groups | Rheumatology Group |
| Kirsi Kalpala | Pfizer, New York, NY, United States | kirsi.kalpala@pfizer.com | Clinical Groups | Rheumatology Group |
| Nan Bing | Pfizer, New York, NY, United States | nan.bing@pfizer.com | Clinical Groups | Rheumatology Group |
| Xinli Hu | Pfizer, New York, NY, United States | xinli.hu@pfizer.com | Clinical Groups | Rheumatology Group |
| Tarja Laitinen | Pirkanmaa Hospital District, Tampere, Finland | tarja.laitinen@pshp.fi | Clinical Groups | Pulmonology Group |
| Margit Pelkonen | Northern Savo Hospital District, Kuopio, Finland | margit.pelkonen@kuh.fi | Clinical Groups | Pulmonology Group |
| Paula Kauppi | Hospital District of Helsinki and Uusimaa, Helsinki, Finland | paula.kauppi@hus.fi | Clinical Groups | Pulmonology Group |
| Hannu Kankaanranta | University of Gothenburg, Gothenburg, Sweden/ Seinäjoki Central Hospital, Seinäjoki, Finland/ Tampere University, Tampere, Finland | hannu.kankaanranta@tuni.fi | Clinical Groups | Pulmonology Group |
| Terttu Harju | Northern Ostrobothnia Hospital District, Oulu, Finland | terttu.harju@oulu.fi | Clinical Groups | Pulmonology Group |
| Riitta Lahesmaa | Hospital District of Southwest Finland, Turku, Finland | rilahes@utu.fi | Clinical Groups | Pulmonology Group |
| Nizar Smaoui | Abbvie, Chicago, IL, United States | nizar.smaoui@abbvie.com | Clinical Groups | Pulmonology Group |
| Glenda Lassi | Astra Zeneca, Cambridge, United Kingdom | glenda.lassi@astrazeneca.com | Clinical Groups | Pulmonology Group |
| Susan Eaton | Biogen, Cambridge, MA, United States | susan.eaton@biogen.com | Clinical Groups | Pulmonology Group |
| Hubert Chen | Genentech, San Francisco, CA, United States | chenh37@gene.com | Clinical Groups | Pulmonology Group |
| Rion Pendergrass | Genentech, San Francisco, CA, United States | penders2@gene.com | Clinical Groups | Pulmonology Group |
| Natalie Bowers | Genentech, San Francisco, CA, United States | bowersn1@gene.com | Clinical Groups | Pulmonology Group |
| Joanna Betts | GlaxoSmithKline, Brentford, United Kingdom | joanna.c.betts@gsk.com | Clinical Groups | Pulmonology Group |
| Kirsi Auro | GlaxoSmithKline, Espoo, Finland | kirsi.m.auro@gsk.com | Clinical Groups | Pulmonology Group |
| Rajashree Mishra | GlaxoSmithKline, Brentford, United Kingdom | rajashree.x.mishra@gsk.com | Clinical Groups | Pulmonology Group |
| Majd Mouded | Novartis, Basel, Switzerland | majd.mouded@novartis.com | Clinical Groups | Pulmonology Group |
| Debby Ngo | Novartis, Basel, Switzerland | debby.ngo@novartis.com | Clinical Groups | Pulmonology Group |
| Teemu Niiranen | Finnish Institute for Health and Welfare (THL), Helsinki, Finland | teemu.niiranen@thl.fi | Clinical Groups | Cardiometabolic Diseases Group |
| Felix Vaura | Finnish Institute for Health and Welfare (THL), Helsinki, Finland | fechva@utu.fi | Clinical Groups | Cardiometabolic Diseases Group |
| Veikko Salomaa | Finnish Institute for Health and Welfare (THL), Helsinki, Finland | veikko.salomaa@thl.fi | Clinical Groups | Cardiometabolic Diseases Group |
| Kaj Metsärinne | Hospital District of Southwest Finland, Turku, Finland | kaj.metsarinne@tyks.fi | Clinical Groups | Cardiometabolic Diseases Group |
| Jenni Aittokallio | Hospital District of Southwest Finland, Turku, Finland | jemato@utu.fi | Clinical Groups | Cardiometabolic Diseases Group |
| Mika Kähönen | Pirkanmaa Hospital District, Tampere, Finland | mika.kahonen@uta.fi | Clinical Groups | Cardiometabolic Diseases Group |
| Jussi Hernesniemi | Pirkanmaa Hospital District, Tampere, Finland | jussi.hernesniemi@tuni.fi | Clinical Groups | Cardiometabolic Diseases Group |
| Daniel Gordin | Hospital District of Helsinki and Uusimaa, Helsinki, Finland | daniel.gordin@hus.fi | Clinical Groups | Cardiometabolic Diseases Group |
| Juha Sinisalo | Hospital District of Helsinki and Uusimaa, Helsinki, Finland | juha.sinisalo@hus.fi | Clinical Groups | Cardiometabolic Diseases Group |
| Marja-Riitta Taskinen | Hospital District of Helsinki and Uusimaa, Helsinki, Finland | marja-riitta.taskinen@helsinki.fi | Clinical Groups | Cardiometabolic Diseases Group |
| Tiinamaija Tuomi | Hospital District of Helsinki and Uusimaa, Helsinki, Finland | tiinamaija.tuomi@hus.fi | Clinical Groups | Cardiometabolic Diseases Group |
| Timo Hiltunen | Hospital District of Helsinki and Uusimaa, Helsinki, Finland | timo.hiltunen@hus.fi | Clinical Groups | Cardiometabolic Diseases Group |
| Jari Laukkanen | Central Finland Health Care District, Jyväskylä, Finland | jari.laukkanen@ksshp.fi | Clinical Groups | Cardiometabolic Diseases Group |
| Amanda Elliott | Institute for Molecular Medicine Finland (FIMM), HiLIFE, University of Helsinki, Helsinki, Finland; Broad Institute, Cambridge, MA, USA and Massachusetts General Hospital, Boston, MA, USA | aelliott@broadinstitute.org | Clinical Groups | Cardiometabolic Diseases Group |
| Mary Pat Reeve | Institute for Molecular Medicine Finland (FIMM), HiLIFE, University of Helsinki, Helsinki, Finland | mary.reeve@helsinki.fi | Clinical Groups | Cardiometabolic Diseases Group |
| Sanni Ruotsalainen | Institute for Molecular Medicine Finland (FIMM), HiLIFE, University of Helsinki, Helsinki, Finland | sanni.ruotsalainen@helsinki.fi | Clinical Groups | Cardiometabolic Diseases Group |
| Benjamin Challis | Astra Zeneca, Cambridge, United Kingdom | benjamin.challis@astrazeneca.com | Clinical Groups | Cardiometabolic Diseases Group |
| Dirk Paul | Astra Zeneca, Cambridge, United Kingdom | dirk.paul@astrazeneca.com | Clinical Groups | Cardiometabolic Diseases Group |
| Julie Hunkapiller | Genentech, San Francisco, CA, United States | hunkapiller.julie@gene.com | Clinical Groups | Cardiometabolic Diseases Group |
| Natalie Bowers | Genentech, San Francisco, CA, United States | bowersn1@gene.com | Clinical Groups | Cardiometabolic Diseases Group |
| Rion Pendergrass | Genentech, San Francisco, CA, United States | penders2@gene.com | Clinical Groups | Cardiometabolic Diseases Group |
| Audrey Chu | GlaxoSmithKline, Brentford, United Kingdom | audrey.y.chu@gsk.com | Clinical Groups | Cardiometabolic Diseases Group |
| Kirsi Auro | GlaxoSmithKline, Espoo, Finland | kirsi.m.auro@gsk.com | Clinical Groups | Cardiometabolic Diseases Group |
| Dermot Reilly | Janssen Research & Development, LLC, Boston, MA, United States | dreill11@its.jnj.com | Clinical Groups | Cardiometabolic Diseases Group |
| Mike Mendelson | Novartis, Boston, MA, United States | mike.mendelson@novartis.com | Clinical Groups | Cardiometabolic Diseases Group |
| Jaakko Parkkinen | Pfizer, New York, NY, United States | jaakko.parkkinen@pfizer.com | Clinical Groups | Cardiometabolic Diseases Group |
| Melissa Miller | Pfizer, New York, NY, United States | melissa.r.miller@pfizer.com | Clinical Groups | Cardiometabolic Diseases Group |
| Tuomo Meretoja | Hospital District of Helsinki and Uusimaa, Helsinki, Finland | tuomo.meretoja@hus.fi | Clinical Groups | Oncology Group |
| Heikki Joensuu | Hospital District of Helsinki and Uusimaa, Helsinki, Finland | heikki.joensuu@hus.fi | Clinical Groups | Oncology Group |
| Olli Carpén | Hospital District of Helsinki and Uusimaa, Helsinki, Finland | olli.carpen@helsinki.fi | Clinical Groups | Oncology Group |
| Johanna Mattson | Hospital District of Helsinki and Uusimaa, Helsinki, Finland | johanna.mattson@hus.fi | Clinical Groups | Oncology Group |
| Eveliina Salminen | Hospital District of Helsinki and Uusimaa, Helsinki, Finland | eveliina.e.salminen@hus.fi | Clinical Groups | Oncology Group |
| Annika Auranen | Pirkanmaa Hospital District , Tampere, Finland | anaura@utu.fi | Clinical Groups | Oncology Group |
| Peeter Karihtala | Northern Ostrobothnia Hospital District, Oulu, Finland | peeter.karihtala@oulu.fi | Clinical Groups | Oncology Group |
| Päivi Auvinen | Northern Savo Hospital District, Kuopio, Finland | paivi.auvinen@kuh.fi | Clinical Groups | Oncology Group |
| Klaus Elenius | Hospital District of Southwest Finland, Turku, Finland | klaus.elenius@utu.fi | Clinical Groups | Oncology Group |
| Johanna Schleutker | Hospital District of Southwest Finland, Turku, Finland | johanna.schleutker@utu.fi | Clinical Groups | Oncology Group |
| Esa Pitkänen | Institute for Molecular Medicine Finland (FIMM), HiLIFE, University of Helsinki, Helsinki, Finland | esa.pitkanen@helsinki.fi | Clinical Groups | Oncology Group |
| Nina Mars | Institute for Molecular Medicine Finland (FIMM), HiLIFE, University of Helsinki, Helsinki, Finland | nina.mars@helsinki.fi | Clinical Groups | Oncology Group |
| Mark Daly | Institute for Molecular Medicine Finland (FIMM), HiLIFE, University of Helsinki, Helsinki, Finland; Broad Institute of MIT and Harvard; Massachusetts General Hospital | mark.daly@helsinki.fi | Clinical Groups | Oncology Group |
| Relja Popovic | Abbvie, Chicago, IL, United States | relja.popovic@abbvie.com | Clinical Groups | Oncology Group |
| Jeffrey Waring | Abbvie, Chicago, IL, United States | jeff.waring@abbvie.com | Clinical Groups | Oncology Group |
| Bridget Riley-Gillis | Abbvie, Chicago, IL, United States | bridget.rileygillis@abbvie.com | Clinical Groups | Oncology Group |
| Anne Lehtonen | Abbvie, Chicago, IL, United States | anne.lehtonen@abbvie.com | Clinical Groups | Oncology Group |
| Jennifer Schutzman | Genentech, San Francisco, CA, United States | schutzman.jennifer@gene.com | Clinical Groups | Oncology Group |
| Julie Hunkapiller | Genentech, San Francisco, CA, United States | hunkapiller.julie@gene.com | Clinical Groups | Oncology Group |
| Natalie Bowers | Genentech, San Francisco, CA, United States | bowersn1@gene.com | Clinical Groups | Oncology Group |
| Rion Pendergrass | Genentech, San Francisco, CA, United States | penders2@gene.com | Clinical Groups | Oncology Group |
| Diptee Kulkarni | GlaxoSmithKline, Brentford, United Kingdom | diptee.a.kulkarni@gsk.com | Clinical Groups | Oncology Group |
| Kirsi Auro | GlaxoSmithKline, Espoo, Finland | kirsi.m.auro@gsk.com | Clinical Groups | Oncology Group |
| Alessandro Porello | Janssen Research & Development, LLC, Spring House, PA, United States | APorrell@ITS.JNJ.com | Clinical Groups | Oncology Group |
| Andrey Loboda | Merck, Kenilworth, NJ, United States | andrey_loboda@merck.com | Clinical Groups | Oncology Group |
| Heli Lehtonen | Pfizer, New York, NY, United States | heli.lehtonen@pfizer.com | Clinical Groups | Oncology Group |
| Stefan McDonough | Pfizer, New York, NY, United States | stefan.McDonough@pfizer.com | Clinical Groups | Oncology Group |
| Sauli Vuoti | Janssen-Cilag Oy, Espoo, Finland | svuoti@its.jnj.com | Clinical Groups | Oncology Group |
| Kai Kaarniranta | Northern Savo Hospital District, Kuopio, Finland | kai.kaarniranta@uef.fi | Clinical Groups | Opthalmology Group |
| Joni A Turunen | Helsinki University Hospital and University of Helsinki, Helsinki, Finland; Eye Genetics Group, Folkhälsan Research Center, Helsinki, Finland | joni.turunen@helsinki.fi | Clinical Groups | Opthalmology Group |
| Terhi Ollila | Hospital District of Helsinki and Uusimaa, Helsinki, Finland | terhi.ollila@hus.fi | Clinical Groups | Opthalmology Group |
| Hannu Uusitalo | Pirkanmaa Hospital District, Tampere, Finland | hannu.uusitalo@tuni.fi | Clinical Groups | Opthalmology Group |
| Juha Karjalainen | Institute for Molecular Medicine Finland (FIMM), HiLIFE, University of Helsinki, Helsinki, Finland | juha.karjalainen@helsinki.fi | Clinical Groups | Opthalmology Group |
| Esa Pitkänen | Institute for Molecular Medicine Finland (FIMM), HiLIFE, University of Helsinki, Helsinki, Finland | esa.pitkanen@helsinki.fi | Clinical Groups | Opthalmology Group |
| Mengzhen Liu | Abbvie, Chicago, IL, United States | mengzhen.liu@abbvie.com | Clinical Groups | Opthalmology Group |
| Heiko Runz | Biogen, Cambridge, MA, United States | heiko.runz@biogen.com | Clinical Groups | Opthalmology Group |
| Stephanie Loomis | Biogen, Cambridge, MA, United States | stephanie.loomis@biogen.com | Clinical Groups | Opthalmology Group |
| Erich Strauss | Genentech, San Francisco, CA, United States | strauss.erich@gene.com | Clinical Groups | Opthalmology Group |
| Natalie Bowers | Genentech, San Francisco, CA, United States | bowersn1@gene.com | Clinical Groups | Opthalmology Group |
| Hao Chen | Genentech, San Francisco, CA, United States | haoc@gene.com | Clinical Groups | Opthalmology Group |
| Rion Pendergrass | Genentech, San Francisco, CA, United States | penders2@gene.com | Clinical Groups | Opthalmology Group |
| Kaisa Tasanen | Northern Ostrobothnia Hospital District, Oulu, Finland | kaisa.tasanen-maatta@oulu.fi | Clinical Groups | Dermatology Group |
| Laura Huilaja | Northern Ostrobothnia Hospital District, Oulu, Finland | laura.huilaja@oulu.fi | Clinical Groups | Dermatology Group |
| Katariina Hannula-Jouppi | Hospital District of Helsinki and Uusimaa, Helsinki, Finland | katariina.hannula-jouppi@hus.fi | Clinical Groups | Dermatology Group |
| Teea Salmi | Pirkanmaa Hospital District, Tampere, Finland | teea.salmi@pshp.fi | Clinical Groups | Dermatology Group |
| Sirkku Peltonen | Hospital District of Southwest Finland, Turku, Finland | sipelto@utu.fi | Clinical Groups | Dermatology Group |
| Leena Koulu | Hospital District of Southwest Finland, Turku, Finland | leena.koulu@tyks.fi | Clinical Groups | Dermatology Group |
| Nizar Smaoui | Abbvie, Chicago, IL, United States | nizar.smaoui@abbvie.com | Clinical Groups | Dermatology Group |
| Fedik Rahimov | Abbvie, Chicago, IL, United States | fedik.rahimov@abbvie.com | Clinical Groups | Dermatology Group |
| Anne Lehtonen | Abbvie, Chicago, IL, United States | anne.lehtonen@abbvie.com | Clinical Groups | Dermatology Group |
| David Choy | Genentech, San Francisco, CA, United States | choy.david@gene.com | Clinical Groups | Dermatology Group |
| Rion Pendergrass | Genentech, San Francisco, CA, United States | penders2@gene.com | Clinical Groups | Dermatology Group |
| Dawn Waterworth | Janssen Research & Development, LLC, Spring House, PA, United States | dwaterwo@its.jnj.com | Clinical Groups | Dermatology Group |
| Kirsi Kalpala | Pfizer, New York, NY, United States | kirsi.kalpala@pfizer.com | Clinical Groups | Dermatology Group |
| Ying Wu | Pfizer, New York, NY, United States | ying.wu3@pfizer.com | Clinical Groups | Dermatology Group |
| Pirkko Pussinen | Hospital District of Helsinki and Uusimaa, Helsinki, Finland | pirkko.pussinen@helsinki.fi | Clinical Groups | Odontology Group |
| Aino Salminen | Hospital District of Helsinki and Uusimaa, Helsinki, Finland | aino.m.salminen@helsinki.fi | Clinical Groups | Odontology Group |
| Tuula Salo | Hospital District of Helsinki and Uusimaa, Helsinki, Finland | tuula.salo@helsinki.fi | Clinical Groups | Odontology Group |
| David Rice | Hospital District of Helsinki and Uusimaa, Helsinki, Finland | david.rice@helsinki.fi | Clinical Groups | Odontology Group |
| Pekka Nieminen | Hospital District of Helsinki and Uusimaa, Helsinki, Finland | pekka.nieminen@helsinki.fi | Clinical Groups | Odontology Group |
| Ulla Palotie | Hospital District of Helsinki and Uusimaa, Helsinki, Finland | ulla.palotie@helsinki.fi | Clinical Groups | Odontology Group |
| Maria Siponen | Northern Savo Hospital District, Kuopio, Finland | maria.siponen@uef.fi | Clinical Groups | Odontology Group |
| Liisa Suominen | Northern Savo Hospital District, Kuopio, Finland | liisa.suominen@uef.fi | Clinical Groups | Odontology Group |
| Päivi Mäntylä | Northern Savo Hospital District, Kuopio, Finland | paivi.mantyla@uef.fi | Clinical Groups | Odontology Group |
| Ulvi Gursoy | Hospital District of Southwest Finland, Turku, Finland | ulvi.gursoy@utu.fi | Clinical Groups | Odontology Group |
| Vuokko Anttonen | Northern Ostrobothnia Hospital District, Oulu, Finland | vuokko.anttonen@oulu.fi | Clinical Groups | Odontology Group |
| Kirsi Sipilä | Research Unit of Oral Health Sciences Faculty of Medicine, University of Oulu, Oulu, Finland; Medical Research Center, Oulu, Oulu University Hospital and University of Oulu, Oulu, Finland | kirsi.sipila@oulu.fi | Clinical Groups | Odontology Group |
| Rion Pendergrass | Genentech, San Francisco, CA, United States | pendergrass.sarah@gene.com | Clinical Groups | Odontology Group |
| Hannele Laivuori | Institute for Molecular Medicine Finland (FIMM), HiLIFE, University of Helsinki, Helsinki, Finland | hannele.laivuori@helsinki.fi | Clinical Groups | Women’s Health and Reproduction Group |
| Venla Kurra | Pirkanmaa Hospital District, Tampere, Finland | venla.kurra@tuni.fi | Clinical Groups | Women’s Health and Reproduction Group |
| Laura Kotaniemi-Talonen | Pirkanmaa Hospital District, Tampere, Finland | laura.kotaniemi-talonen@tuni.fi | Clinical Groups | Women’s Health and Reproduction Group |
| Oskari Heikinheimo | Hospital District of Helsinki and Uusimaa, Helsinki, Finland | oskari.heikinheimo@helsinki.fi | Clinical Groups | Women’s Health and Reproduction Group |
| Ilkka Kalliala | Hospital District of Helsinki and Uusimaa, Helsinki, Finland | ilkka.kalliala@hus.fi | Clinical Groups | Women’s Health and Reproduction Group |
| Lauri Aaltonen | Hospital District of Helsinki and Uusimaa, Helsinki, Finland | lauri.aaltonen@helsinki.fi | Clinical Groups | Women’s Health and Reproduction Group |
| Varpu Jokimaa | Hospital District of Southwest Finland, Turku, Finland | varpu.jokimaa@utu.fi | Clinical Groups | Women’s Health and Reproduction Group |
| Johannes Kettunen | Northern Ostrobothnia Hospital District, Oulu, Finland | Johannes.Kettunen@oulu.fi | Clinical Groups | Women’s Health and Reproduction Group |
| Marja Vääräsmäki | Northern Ostrobothnia Hospital District, Oulu, Finland | marja.vaarasmaki@oulu.fi | Clinical Groups | Women’s Health and Reproduction Group |
| Outi Uimari | Northern Ostrobothnia Hospital District, Oulu, Finland | outi.uimari@oulu.fi | Clinical Groups | Women’s Health and Reproduction Group |
| Laure Morin-Papunen | Northern Ostrobothnia Hospital District, Oulu, Finland | lmp@cc.oulu.fi | Clinical Groups | Women’s Health and Reproduction Group |
| Maarit Niinimäki | Northern Ostrobothnia Hospital District, Oulu, Finland | maarit.niinimaki@oulu.fi | Clinical Groups | Women’s Health and Reproduction Group |
| Terhi Piltonen | Northern Ostrobothnia Hospital District, Oulu, Finland | terhi.piltonen@oulu.fi | Clinical Groups | Women’s Health and Reproduction Group |
| Katja Kivinen | Institute for Molecular Medicine Finland (FIMM), HiLIFE, University of Helsinki, Helsinki, Finland | katja.kivinen@helsinki.fi | Clinical Groups | Women’s Health and Reproduction Group |
| Elisabeth Widen | Institute for Molecular Medicine Finland (FIMM), HiLIFE, University of Helsinki, Helsinki, Finland | elisabeth.widen@helsinki.fi | Clinical Groups | Women’s Health and Reproduction Group |
| Taru Tukiainen | Institute for Molecular Medicine Finland (FIMM), HiLIFE, University of Helsinki, Helsinki, Finland | taru.tukiainen@helsinki.fi | Clinical Groups | Women’s Health and Reproduction Group |
| Mary Pat Reeve | Institute for Molecular Medicine Finland (FIMM), HiLIFE, University of Helsinki, Helsinki, Finland | mary.reeve@helsinki.fi | Clinical Groups | Women’s Health and Reproduction Group |
| Mark Daly | Institute for Molecular Medicine Finland (FIMM), HiLIFE, University of Helsinki, Helsinki, Finland; Broad Institute of MIT and Harvard; Massachusetts General Hospital | mark.daly@helsinki.fi | Clinical Groups | Women’s Health and Reproduction Group |
| Niko Välimäki | University of Helsinki, Helsinki, Finland | niko.valimaki@helsinki.fi | Clinical Groups | Women’s Health and Reproduction Group |
| Eija Laakkonen | University of Jyväskylä, Jyväskylä, Finland | eija.k.laakkonen@jyu.fi | Clinical Groups | Women’s Health and Reproduction Group |
| Jaakko Tyrmi | University of Oulu, Oulu, Finland / University of Tampere, Tampere, Finland | jaakko.tyrmi@oulu.fi | Clinical Groups | Women’s Health and Reproduction Group |
| Heidi Silven | University of Oulu, Oulu, Finland | heidi.silven@student.oulu.fi | Clinical Groups | Women’s Health and Reproduction Group |
| Eeva Sliz | University of Oulu, Oulu, Finland | eeva.sliz@oulu.fi | Clinical Groups | Women’s Health and Reproduction Group |
| Riikka Arffman | University of Oulu, Oulu, Finland | riikka.arffman@oulu.fi | Clinical Groups | Women’s Health and Reproduction Group |
| Susanna Savukoski | University of Oulu, Oulu, Finland | susanna.savukoski@oulu.fi | Clinical Groups | Women’s Health and Reproduction Group |
| Triin Laisk | Estonian biobank, Tartu, Estonia | triin.laisk@ut.ee | Clinical Groups | Women’s Health and Reproduction Group |
| Natalia Pujol | Estonian biobank, Tartu, Estonia | natalia.pujolgualdo@oulu.fi | Clinical Groups | Women’s Health and Reproduction Group |
| Mengzhen Liu | Abbvie, Chicago, IL, United States | mengzhen.liu@abbvie.com | Clinical Groups | Women’s Health and Reproduction Group |
| Bridget Riley-Gillis | Abbvie, Chicago, IL, United States | bridget.rileygillis@abbvie.com | Clinical Groups | Women’s Health and Reproduction Group |
| Rion Pendergrass | Genentech, San Francisco, CA, United States | penders2@gene.com | Clinical Groups | Women’s Health and Reproduction Group |
| Janet Kumar | GlaxoSmithKline, Collegeville, PA, United States | janet.x.kumar@gsk.com | Clinical Groups | Women’s Health and Reproduction Group |
| Kirsi Auro | GlaxoSmithKline, Espoo, Finland | kirsi.m.auro@gsk.com | Clinical Groups | Women’s Health and Reproduction Group |
| Iiris Hovatta | University of Helsinki, Finland | iiris.hovatta@helsinki.fi | Clinical Groups | Depression group |
| Chia-Yen Chen | Biogen, Cambridge, MA, United States | chiayen.chen@biogen.com | Clinical Groups | Depression group |
| Erkki Isometsä | Hospital District of Helsinki and Uusimaa, Helsinki, Finland | erkki.isometsa@hus.fi | Clinical Groups | Depression group |
| Hanna Ollila | Institute for Molecular Medicine Finland (FIMM), HiLIFE, University of Helsinki, Helsinki, Finland | hanna.m.ollila@helsinki.fi | Clinical Groups | Depression group |
| Jaana Suvisaari | Finnish Institute for Health and Welfare (THL), Helsinki, Finland | jaana.suvisaari@thl.fi | Clinical Groups | Depression group |
| Thomas Damm Als | Aarhus University, Denmark | tda@biomed.au.dk | Clinical Groups | Depression group |
| Antti Mäkitie | Department of Otorhinolaryngology - Head and Neck Surgery, University of Helsinki and Helsinki University Hospital, Helsinki, Finland | antti.makitie@helsinki.fi | Clinical Groups | ENT (ear, nose and throath) Group |
| Argyro Bizaki-Vallaskangas | Pirkanmaa Hospital District, Tampere, Finland | argyro.bizaki-vallaskangas@tuni.fi | Clinical Groups | ENT (ear, nose and throath) Group |
| Sanna Toppila-Salmi | University of Helsinki, Finland | sanna.salmi@helsinki.fi | Clinical Groups | ENT (ear, nose and throath) Group |
| Tytti Willberg | Hospital District of Southwest Finland, Turku, Finland | tytti.willberg@tyks.fi | Clinical Groups | ENT (ear, nose and throath) Group |
| Elmo Saarentaus | Institute for Molecular Medicine Finland (FIMM), HiLIFE, University of Helsinki, Helsinki, Finland | elmo.saarentaus@helsinki.fi | Clinical Groups | ENT (ear, nose and throath) Group |
| Antti Aarnisalo | Hospital District of Helsinki and Uusimaa, Helsinki, Finland | antti.aarnisalo@hus.fi | Clinical Groups | ENT (ear, nose and throath) Group |
| Eveliina Salminen | Hospital District of Helsinki and Uusimaa, Helsinki, Finland | eveliina.e.salminen@hus.fi | Clinical Groups | ENT (ear, nose and throath) Group |
| Elisa Rahikkala | Northern Ostrobothnia Hospital District, Oulu, Finland | elisa.rahikkala@ppshp.fi | Clinical Groups | ENT (ear, nose and throath) Group |
| Johannes Kettunen | Northern Ostrobothnia Hospital District, Oulu, Finland | johannes.kettunen@oulu.fi | Clinical Groups | ENT (ear, nose and throath) Group |
| Kristiina Aittomäki | Department of Medical Genetics, Helsinki University Central Hospital, Helsinki, Finland | kristiina.aittomaki@helsinki.fi | Clinical Groups | POI (premature ovarian failure) Group |
| Fredrik Åberg | Transplantation and Liver Surgery Clinic, Helsinki University Hospital, Helsinki University, Helsinki, Finland | fredrik.aberg@helsinki.fi | Clinical Groups | LiverScore Group |
| Mitja Kurki | Institute for Molecular Medicine Finland (FIMM), HiLIFE, University of Helsinki, Helsinki, Finland; Broad Institute, Cambridge, MA, United States | mkurki@broadinstitute.org | FinnGen Analysis working group | FinnGen Analysis working group |
| Samuli Ripatti | Institute for Molecular Medicine Finland (FIMM), HiLIFE, University of Helsinki, Helsinki, Finland | samuli.ripatti@helsinki.fi | FinnGen Analysis working group | FinnGen Analysis working group |
| Mark Daly | Institute for Molecular Medicine, Finland (FIMM), HiLIFE, University of Helsinki, Helsinki, Finland; Broad Institute of MIT and Harvard; Massachusetts General Hospital | mark.daly@helsinki.fi | FinnGen Analysis working group | FinnGen Analysis working group |
| Juha Karjalainen | Institute for Molecular Medicine Finland (FIMM), HiLIFE, University of Helsinki, Helsinki, Finland | juha.karjalainen@helsinki.fi | FinnGen Analysis working group | FinnGen Analysis working group |
| Aki Havulinna | Institute for Molecular Medicine Finland (FIMM), HiLIFE, University of Helsinki, Helsinki, Finland; Finnish Institute for Health and Welfare (THL), Helsinki, Finland | aki.havulinna@helsinki.fi | FinnGen Analysis working group | FinnGen Analysis working group |
| Juha Mehtonen | Institute for Molecular Medicine Finland (FIMM), HiLIFE, University of Helsinki, Helsinki, Finland | juha.mehtonen@helsinki.fi | FinnGen Analysis working group | FinnGen Analysis working group |
| Priit Palta | Institute for Molecular Medicine Finland (FIMM), HiLIFE, University of Helsinki, Helsinki, Finland | priit.palta@helsinki.fi | FinnGen Analysis working group | FinnGen Analysis working group |
| Shabbeer Hassan | Institute for Molecular Medicine Finland (FIMM), HiLIFE, University of Helsinki, Helsinki, Finland | shabbeer.hassan@helsinki.fi | FinnGen Analysis working group | FinnGen Analysis working group |
| Pietro Della Briotta Parolo | Institute for Molecular Medicine Finland (FIMM), HiLIFE, University of Helsinki, Helsinki, Finland | pietro.dellabriottaparolo@helsinki.fi | FinnGen Analysis working group | FinnGen Analysis working group |
| Wei Zhou | Broad Institute, Cambridge, MA, United States | wzhou@broadinstitute.org | FinnGen Analysis working group | FinnGen Analysis working group |
| Mutaamba Maasha | Broad Institute, Cambridge, MA, United States | mmaasha@broadinstitute.org | FinnGen Analysis working group | FinnGen Analysis working group |
| Shabbeer Hassan | Institute for Molecular Medicine Finland (FIMM), HiLIFE, University of Helsinki, Helsinki, Finland | shabbeer.hassan@helsinki.fi | FinnGen Analysis working group | FinnGen Analysis working group |
| Susanna Lemmelä | Institute for Molecular Medicine Finland (FIMM), HiLIFE, University of Helsinki, Helsinki, Finland | susanna.lemmela@helsinki.fi | FinnGen Analysis working group | FinnGen Analysis working group |
| Manuel Rivas | University of Stanford, Stanford, CA, United States | mrivas@stanford.edu | FinnGen Analysis working group | FinnGen Analysis working group |
| Mari E. Niemi | Institute for Molecular Medicine Finland (FIMM), HiLIFE, University of Helsinki, Helsinki, Finland | mari.e.niemi@helsinki.fi | FinnGen Analysis working group | FinnGen Analysis working group |
| Aarno Palotie | Institute for Molecular Medicine Finland (FIMM), HiLIFE, University of Helsinki, Helsinki, Finland | aarno.palotie@helsinki.fi | FinnGen Analysis working group | FinnGen Analysis working group |
| Aoxing Liu | Institute for Molecular Medicine Finland (FIMM), HiLIFE, University of Helsinki, Helsinki, Finland | aoxing.liu@helsinki.fi | FinnGen Analysis working group | FinnGen Analysis working group |
| Arto Lehisto | Institute for Molecular Medicine Finland (FIMM), HiLIFE, University of Helsinki, Helsinki, Finland | arto.lehisto@helsinki.fi | FinnGen Analysis working group | FinnGen Analysis working group |
| Andrea Ganna | Institute for Molecular Medicine Finland (FIMM), HiLIFE, University of Helsinki, Helsinki, Finland | aganna@broadinstitute.org | FinnGen Analysis working group | FinnGen Analysis working group |
| Vincent Llorens | Institute for Molecular Medicine Finland (FIMM), HiLIFE, University of Helsinki, Helsinki, Finland | vincent.llorens@helsinki.fi | FinnGen Analysis working group | FinnGen Analysis working group |
| Hannele Laivuori | Institute for Molecular Medicine Finland (FIMM), HiLIFE, University of Helsinki, Helsinki, Finland | hannele.laivuori@helsinki.fi | FinnGen Analysis working group | FinnGen Analysis working group |
| Taru Tukiainen | Institute for Molecular Medicine Finland (FIMM), HiLIFE, University of Helsinki, Helsinki, Finland | taru.tukiainen@helsinki.fi | FinnGen Analysis working group | FinnGen Analysis working group |
| Mary Pat Reeve | Institute for Molecular Medicine Finland (FIMM), HiLIFE, University of Helsinki, Helsinki, Finland | mary.reeve@helsinki.fi | FinnGen Analysis working group | FinnGen Analysis working group |
| Henrike Heyne | Institute for Molecular Medicine Finland (FIMM), HiLIFE, University of Helsinki, Helsinki, Finland | hheyne@broadinstitute.org | FinnGen Analysis working group | FinnGen Analysis working group |
| Nina Mars | Institute for Molecular Medicine Finland (FIMM), HiLIFE, University of Helsinki, Helsinki, Finland | nina.mars@helsinki.fi | FinnGen Analysis working group | FinnGen Analysis working group |
| Joel Rämö | Institute for Molecular Medicine Finland (FIMM), HiLIFE, University of Helsinki, Helsinki, Finland | joel.ramo@helsinki.fi | FinnGen Analysis working group | FinnGen Analysis working group |
| Elmo Saarentaus | Institute for Molecular Medicine Finland (FIMM), HiLIFE, University of Helsinki, Helsinki, Finland | elmo.saarentaus@helsinki.fi | FinnGen Analysis working group | FinnGen Analysis working group |
| Hanna Ollila | Institute for Molecular Medicine Finland (FIMM), HiLIFE, University of Helsinki, Helsinki, Finland | hanna.m.ollila@helsinki.fi | FinnGen Analysis working group | FinnGen Analysis working group |
| Rodos Rodosthenous | Institute for Molecular Medicine Finland (FIMM), HiLIFE, University of Helsinki, Helsinki, Finland | rodos.rodosthenous@helsinki.fi | FinnGen Analysis working group | FinnGen Analysis working group |
| Satu Strausz | Institute for Molecular Medicine Finland (FIMM), HiLIFE, University of Helsinki, Helsinki, Finland | satu.strausz@helsinki.fi | FinnGen Analysis working group | FinnGen Analysis working group |
| Tuula Palotie | University of Helsinki and Hospital District of Helsinki and Uusimaa, Helsinki, Finland | tuula.palotie@helsinki.fi | FinnGen Analysis working group | FinnGen Analysis working group |
| Kimmo Palin | University of Helsinki, Helsinki, Finland | kimmo.palin@helsinki.fi | FinnGen Analysis working group | FinnGen Analysis working group |
| Javier Garcia-Tabuenca | University of Tampere, Tampere, Finland | javier.graciatabuenca@tuni.fi | FinnGen Analysis working group | FinnGen Analysis working group |
| Harri Siirtola | University of Tampere, Tampere, Finland | harri.siirtola@tuni.fi | FinnGen Analysis working group | FinnGen Analysis working group |
| Tuomo Kiiskinen | Institute for Molecular Medicine Finland (FIMM), HiLIFE, University of Helsinki, Helsinki, Finland | tuomo.kiiskinen@helsinki.fi | FinnGen Analysis working group | FinnGen Analysis working group |
| Jiwoo Lee | Institute for Molecular Medicine Finland (FIMM), HiLIFE, University of Helsinki, Helsinki, Finland; Broad Institute, Cambridge, MA, United States | jiwoo.lee@helsinki.fi | FinnGen Analysis working group | FinnGen Analysis working group |
| Kristin Tsuo | Institute for Molecular Medicine Finland (FIMM), HiLIFE, University of Helsinki, Helsinki, Finland; Broad Institute, Cambridge, MA, United States | kristintsuo@fas.harvard.edu | FinnGen Analysis working group | FinnGen Analysis working group |
| Amanda Elliott | Institute for Molecular Medicine Finland (FIMM), HiLIFE, University of Helsinki, Helsinki, Finland; Broad Institute, Cambridge, MA, USA and Massachusetts General Hospital, Boston, MA, USA | aelliott@broadinstitute.org | FinnGen Analysis working group | FinnGen Analysis working group |
| Kati Kristiansson | THL Biobank / Finnish Institute for Health and Welfare (THL), Helsinki, Finland | kati.kristiansson@thl.fi | FinnGen Analysis working group | FinnGen Analysis working group |
| Mikko Arvas | Finnish Red Cross Blood Service / Finnish Hematology Registry and Clinical Biobank, Helsinki, Finland | mikko.arvas@veripalvelu.fi | FinnGen Analysis working group | FinnGen Analysis working group |
| Kati Hyvärinen | Finnish Red Cross Blood Service, Helsinki, Finland | kati.hyvarinen@veripalvelu.fi | FinnGen Analysis working group | FinnGen Analysis working group |
| Jarmo Ritari | Finnish Red Cross Blood Service, Helsinki, Finland | jarmo.ritari@veripalvelu.fi | FinnGen Analysis working group | FinnGen Analysis working group |
| Olli Carpén | Helsinki Biobank / Helsinki University and Hospital District of Helsinki and Uusimaa, Helsinki | olli.carpen@helsinki.fi | FinnGen Analysis working group | FinnGen Analysis working group |
| Johannes Kettunen | Northern Finland Biobank Borealis / University of Oulu / Northern Ostrobothnia Hospital District, Oulu, Finland | johannes.kettunen@oulu.fi | FinnGen Analysis working group | FinnGen Analysis working group |
| Katri Pylkäs | University of Oulu, Oulu, Finland | katri.pylkas@oulu.fi | FinnGen Analysis working group | FinnGen Analysis working group |
| Eeva Sliz | University of Oulu, Oulu, Finland | eeva.sliz@oulu.fi | FinnGen Analysis working group | FinnGen Analysis working group |
| Minna Karjalainen | University of Oulu, Oulu, Finland | minna.k.karjalainen@oulu.fi | FinnGen Analysis working group | FinnGen Analysis working group |
| Tuomo Mantere | Northern Finland Biobank Borealis / University of Oulu / Northern Ostrobothnia Hospital District, Oulu, Finland | tuomo.mantere@oulu.fi | FinnGen Analysis working group | FinnGen Analysis working group |
| Eeva Kangasniemi | Finnish Clinical Biobank Tampere / University of Tampere / Pirkanmaa Hospital District, Tampere, Finland | eeva.kangasniemi@pshp.fi | FinnGen Analysis working group | FinnGen Analysis working group |
| Sami Heikkinen | University of Eastern Finland, Kuopio, Finland | sami.heikkinen@uef.fi | FinnGen Analysis working group | FinnGen Analysis working group |
| Arto Mannermaa | Biobank of Eastern Finland / University of Eastern Finland / Northern Savo Hospital District, Kuopio, Finland | arto.mannermaa@uef.fi | FinnGen Analysis working group | FinnGen Analysis working group |
| Eija Laakkonen | University of Jyväskylä, Jyväskylä, Finland | eija.k.laakkonen@jyu.fi | FinnGen Analysis working group | FinnGen Analysis working group |
| Nina Pitkänen | Auria Biobank / University of Turku / Hospital District of Southwest Finland, Turku, Finland | Niina.Pitkanen@tyks.fi | FinnGen Analysis working group | FinnGen Analysis working group |
| Samuel Lessard | Translational Sciences, Sanofi R&D, Framingham, MA, USA | samuel.lessard@sanofi.com | FinnGen Analysis working group | FinnGen Analysis working group |
| Clément Chatelain | Translational Sciences, Sanofi R&D, Framingham, MA, USA | clement.chatelain@sanofi.com | FinnGen Analysis working group | FinnGen Analysis working group |
| Perttu Terho | Auria Biobank / University of Turku / Hospital District of Southwest Finland, Turku, Finland | perttu.terho@tyks.fi | Biobank directors | Biobank directors |
| Sirpa Soini | THL Biobank / Finnish Institute for Health and Welfare (THL), Helsinki, Finland | sirpa.soini@thl.fi | Biobank directors | Biobank directors |
| Jukka Partanen | Finnish Red Cross Blood Service / Finnish Hematology Registry and Clinical Biobank, Helsinki, Finland | jukka.partanen@veripalvelu.fi | Biobank directors | Biobank directors |
| Eero Punkka | Helsinki Biobank / Helsinki University and Hospital District of Helsinki and Uusimaa, Helsinki | eero.punkka@hus.fi | Biobank directors | Biobank directors |
| Raisa Serpi | Northern Finland Biobank Borealis / University of Oulu / Northern Ostrobothnia Hospital District, Oulu, Finland | raisa.serpi@ppshp.fi | Biobank directors | Biobank directors |
| Sanna Siltanen | Finnish Clinical Biobank Tampere / University of Tampere / Pirkanmaa Hospital District, Tampere, Finland | sanna.siltanen@pshp.fi | Biobank directors | Biobank directors |
| Veli-Matti Kosma | Biobank of Eastern Finland / University of Eastern Finland / Northern Savo Hospital District, Kuopio, Finland | veli-matti.kosma@uef.fi | Biobank directors | Biobank directors |
| Teijo Kuopio | Central Finland Biobank / University of Jyväskylä / Central Finland Health Care District, Jyväskylä, Finland | teijo.kuopio@ksshp.fi | Biobank directors | Biobank directors |
| Anu Jalanko | Institute for Molecular Medicine Finland (FIMM), HiLIFE, University of Helsinki, Helsinki, Finland | anu.jalanko@helsinki.fi | FinnGen Teams | Administration |
| Huei-Yi Shen | Institute for Molecular Medicine Finland (FIMM), HiLIFE, University of Helsinki, Helsinki, Finland | huei-yi.shen@helsinki.fi | FinnGen Teams | Administration |
| Risto Kajanne | Institute for Molecular Medicine Finland (FIMM), HiLIFE, University of Helsinki, Helsinki, Finland | risto.kajanne@helsinki.fi | FinnGen Teams | Administration |
| Mervi Aavikko | Institute for Molecular Medicine Finland (FIMM), HiLIFE, University of Helsinki, Helsinki, Finland | mervi.aavikko@helsinki.fi | FinnGen Teams | Administration |
| Henna Palin | Finnish Clinical Biobank Tampere / University of Tampere / Pirkanmaa Hospital District, Tampere, Finland | henna.palin@pshp.fi | FinnGen Teams | Administration |
| Malla-Maria Linna | Helsinki Biobank / Helsinki University and Hospital District of Helsinki and Uusimaa, Helsinki | malla-maria.linna@hus.fi | FinnGen Teams | Administration |
| Mitja Kurki | Institute for Molecular Medicine Finland (FIMM), HiLIFE, University of Helsinki, Helsinki, Finland; Broad Institute, Cambridge, MA, United States | mkurki@broadinstitute.org | FinnGen Teams | Analysis |
| Juha Karjalainen | Institute for Molecular Medicine Finland (FIMM), HiLIFE, University of Helsinki, Helsinki, Finland | juha.karjalainen@helsinki.fi | FinnGen Teams | Analysis |
| Pietro Della Briotta Parolo | Institute for Molecular Medicine Finland (FIMM), HiLIFE, University of Helsinki, Helsinki, Finland | pietro.dellabriottaparolo@helsinki.fi | FinnGen Teams | Analysis |
| Arto Lehisto | Institute for Molecular Medicine Finland (FIMM), HiLIFE, University of Helsinki, Helsinki, Finland | arto.lehisto@helsinki.fi | FinnGen Teams | Analysis |
| Juha Mehtonen | Institute for Molecular Medicine Finland (FIMM), HiLIFE, University of Helsinki, Helsinki, Finland | juha.mehtonen@helsinki.fi | FinnGen Teams | Analysis |
| Wei Zhou | Broad Institute, Cambridge, MA, United States | wzhou@broadinstitute.org | FinnGen Teams | Analysis |
| Masahiro Kanai | Broad Institute, Cambridge, MA, United States | mkanai@broadinstitute.org | FinnGen Teams | Analysis |
| Mutaamba Maasha | Broad Institute, Cambridge, MA, United States | mmaasha@broadinstitute.org | FinnGen Teams | Analysis |
| Hannele Laivuori | Institute for Molecular Medicine Finland (FIMM), HiLIFE, University of Helsinki, Helsinki, Finland | hannele.laivuori@helsinki.fi | FinnGen Teams | Clinical Endpoint Development |
| Aki Havulinna | Institute for Molecular Medicine Finland (FIMM), HiLIFE, University of Helsinki, Helsinki, Finland; Finnish Institute for Health and Welfare (THL), Helsinki, Finland | aki.havulinna@helsinki.fi | FinnGen Teams | Clinical Endpoint Development |
| Susanna Lemmelä | Institute for Molecular Medicine Finland (FIMM), HiLIFE, University of Helsinki, Helsinki, Finland | susanna.lemmela@helsinki.fi | FinnGen Teams | Clinical Endpoint Development |
| Tuomo Kiiskinen | Institute for Molecular Medicine Finland (FIMM), HiLIFE, University of Helsinki, Helsinki, Finland | tuomo.kiiskinen@helsinki.fi | FinnGen Teams | Clinical Endpoint Development |
| L. Elisa Lahtela | Institute for Molecular Medicine Finland (FIMM), HiLIFE, University of Helsinki, Helsinki, Finland | laura.lahtela@helsinki.fi | FinnGen Teams | Clinical Endpoint Development |
| Mari Kaunisto | Institute for Molecular Medicine Finland (FIMM), HiLIFE, University of Helsinki, Helsinki, Finland | mari.kaunisto@helsinki.fi | FinnGen Teams | Communication |
| Elina Kilpeläinen | Institute for Molecular Medicine Finland (FIMM), HiLIFE, University of Helsinki, Helsinki, Finland | elina.kilpelainen@helsinki.fi | FinnGen Teams | E-Science |
| Timo P. Sipilä | Institute for Molecular Medicine Finland (FIMM), HiLIFE, University of Helsinki, Helsinki, Finland | timo.p.sipila@helsinki.fi | FinnGen Teams | E-Science |
| Oluwaseun Alexander Dada | Institute for Molecular Medicine Finland (FIMM), HiLIFE, University of Helsinki, Helsinki, Finland | alexander.dada@helsinki.fi | FinnGen Teams | E-Science |
| Awaisa Ghazal | Institute for Molecular Medicine Finland (FIMM), HiLIFE, University of Helsinki, Helsinki, Finland | awaisa.ghazal@helsinki.fi | FinnGen Teams | E-Science |
| Anastasia Kytölä | Institute for Molecular Medicine Finland (FIMM), HiLIFE, University of Helsinki, Helsinki, Finland | anastasia.shcherban@helsinki.fi | FinnGen Teams | E-Science |
| Rigbe Weldatsadik | Institute for Molecular Medicine Finland (FIMM), HiLIFE, University of Helsinki, Helsinki, Finland | rigbe.weldatsadik@helsinki.fi | FinnGen Teams | E-Science |
| Sanni Ruotsalainen | Institute for Molecular Medicine Finland (FIMM), HiLIFE, University of Helsinki, Helsinki, Finland | sanni.ruotsalainen@helsinki.fi | FinnGen Teams | E-Science |
| Kati Donner | Institute for Molecular Medicine Finland (FIMM), HiLIFE, University of Helsinki, Helsinki, Finland | kati.donner@helsinki.fi | FinnGen Teams | Genotyping |
| Timo P. Sipilä | Institute for Molecular Medicine Finland (FIMM), HiLIFE, University of Helsinki, Helsinki, Finland | timo.p.sipila@helsinki.fi | FinnGen Teams | Genotyping |
| Anu Loukola | Helsinki Biobank / Helsinki University and Hospital District of Helsinki and Uusimaa, Helsinki | anu.loukola@hus.fi | FinnGen Teams | Sample Collection Coordination |
| Päivi Laiho | THL Biobank / Finnish Institute for Health and Welfare (THL), Helsinki, Finland | paivi.laiho@thl.fi | FinnGen Teams | Sample Logistics |
| Tuuli Sistonen | THL Biobank / Finnish Institute for Health and Welfare (THL), Helsinki, Finland | tuuli.sistonen@thl.fi | FinnGen Teams | Sample Logistics |
| Essi Kaiharju | THL Biobank / Finnish Institute for Health and Welfare (THL), Helsinki, Finland | essi.kaiharju@thl.fi | FinnGen Teams | Sample Logistics |
| Markku Laukkanen | THL Biobank / Finnish Institute for Health and Welfare (THL), Helsinki, Finland | markku.laukkanen@thl.fi | FinnGen Teams | Sample Logistics |
| Elina Järvensivu | THL Biobank / Finnish Institute for Health and Welfare (THL), Helsinki, Finland | elina.jarvensivu@thl.fi | FinnGen Teams | Sample Logistics |
| Sini Lähteenmäki | THL Biobank / Finnish Institute for Health and Welfare (THL), Helsinki, Finland | sini.lahteenmaki@thl.fi | FinnGen Teams | Sample Logistics |
| Lotta Männikkö | THL Biobank / Finnish Institute for Health and Welfare (THL), Helsinki, Finland | lotta.mannikko@thl.fi | FinnGen Teams | Sample Logistics |
| Regis Wong | THL Biobank / Finnish Institute for Health and Welfare (THL), Helsinki, Finland | regis.wong@thl.fi | FinnGen Teams | Sample Logistics |
| Auli Toivola | THL Biobank / Finnish Institute for Health and Welfare (THL), Helsinki, Finland | auli.toivola@thl.fi | FinnGen Teams | Sample Logistics |
| Minna Brunfeldt | THL Biobank / Finnish Institute for Health and Welfare (THL), Helsinki, Finland | minna.brunfeldt@thl.fi | FinnGen Teams | Registry Data Operations |
| Hannele Mattsson | THL Biobank / Finnish Institute for Health and Welfare (THL), Helsinki, Finland | hannele.mattsson@thl.fi | FinnGen Teams | Registry Data Operations |
| Kati Kristiansson | THL Biobank / Finnish Institute for Health and Welfare (THL), Helsinki, Finland | kati.kristiansson@thl.fi | FinnGen Teams | Registry Data Operations |
| Susanna Lemmelä | Institute for Molecular Medicine Finland (FIMM), HiLIFE, University of Helsinki, Helsinki, Finland | susanna.lemmela@helsinki.fi | FinnGen Teams | Registry Data Operations |
| Sami Koskelainen | THL Biobank / Finnish Institute for Health and Welfare (THL), Helsinki, Finland | sami.koskelainen@thl.fi | FinnGen Teams | Registry Data Operations |
| Tero Hiekkalinna | THL Biobank / Finnish Institute for Health and Welfare (THL), Helsinki, Finland | tero.hiekkalinna@helsinki.fi | FinnGen Teams | Registry Data Operations |
| Teemu Paajanen | THL Biobank / Finnish Institute for Health and Welfare (THL), Helsinki, Finland | teemu.paajanen@thl.fi | FinnGen Teams | Registry Data Operations |
| Priit Palta | Institute for Molecular Medicine Finland (FIMM), HiLIFE, University of Helsinki, Helsinki, Finland | priit.palta@helsinki.fi | FinnGen Teams | Sequencing Informatics |
| Kalle Pärn | Institute for Molecular Medicine Finland (FIMM), HiLIFE, University of Helsinki, Helsinki, Finland | kalle.parn@helsinki.fi | FinnGen Teams | Sequencing Informatics |
| Mart Kals | Institute for Molecular Medicine Finland (FIMM), HiLIFE, University of Helsinki, Helsinki, Finland | mart.kals@helsinki.fi | FinnGen Teams | Sequencing Informatics |
| Shuang Luo | Institute for Molecular Medicine Finland (FIMM), HiLIFE, University of Helsinki, Helsinki, Finland | shuang.luo@helsinki.fi | FinnGen Teams | Sequencing Informatics |
| Tarja Laitinen | Pirkanmaa Hospital District, Tampere, Finland | tarja.laitinen@pshp.fi | FinnGen Teams | Trajectory |
| Mary Pat Reeve | Institute for Molecular Medicine Finland (FIMM), HiLIFE, University of Helsinki, Helsinki, Finland | mary.reeve@helsinki.fi | FinnGen Teams | Trajectory |
| Shanmukha Sampath Padmanabhuni | Institute for Molecular Medicine Finland (FIMM), HiLIFE, University of Helsinki, Helsinki, Finland | sam.padmanabhuni@helsinki.fi | FinnGen Teams | Trajectory |
| Marianna Niemi | University of Tampere, Tampere, Finland | marianna.niemi@tuni.fi | FinnGen Teams | Trajectory |
| Harri Siirtola | University of Tampere, Tampere, Finland | harri.siirtola@tuni.fi | FinnGen Teams | Trajectory |
| Javier Gracia-Tabuenca | University of Tampere, Tampere, Finland | javier.graciatabuenca@tuni.fi | FinnGen Teams | Trajectory |
| Mika Helminen | University of Tampere, Tampere, Finland | mika.helminen@tuni.fi | FinnGen Teams | Trajectory |
| Tiina Luukkaala | University of Tampere, Tampere, Finland | tiina.luukkaala@tuni.fi | FinnGen Teams | Trajectory |
| Iida Vähätalo | University of Tampere, Tampere, Finland | iida.vahatalo@epshp.fi | FinnGen Teams | Trajectory |
| Jyrki Pitkänen | Institute for Molecular Medicine Finland (FIMM), HiLIFE, University of Helsinki, Helsinki, Finland | jyrki.pitkanen@helsinki.fi | FinnGen Teams | Data protection officer |
| Marco Hautalahti | Finnish Biobank Cooperative - FINBB | marco.hautalahti@finbb.fi | FinnGen Teams | FINBB - Finnish biobank cooperative |
| Johanna Mäkelä | Finnish Biobank Cooperative - FINBB | johanna.makela@finbb.fi | FinnGen Teams | FINBB - Finnish biobank cooperative |
| Sarah Smith | Finnish Biobank Cooperative - FINBB | sarah.smith@finbb.fi | FinnGen Teams | FINBB - Finnish biobank cooperative |
| Tom Southerington | Finnish Biobank Cooperative - FINBB | tom.southerington@finbb.fi | FinnGen Teams | FINBB - Finnish biobank cooperative |
| FinnGen |  |  |  |  |
